# Elastic Fibers/Fabrics for Wearables and Bioelectronics

**DOI:** 10.1002/advs.202203808

**Published:** 2022-10-17

**Authors:** Yufan Zhang, Jiahui Zhou, Yue Zhang, Desuo Zhang, Ken Tye Yong, Jiaqing Xiong

**Affiliations:** ^1^ Innovation Center for Textile Science and Technology Donghua University Shanghai 201620 China; ^2^ College of Textile and Clothing Engineering Soochow University Suzhou 215123 China; ^3^ School of Biomedical Engineering The University of Sydney Sydney New South Wales 2006 Australia

**Keywords:** bioelectronics, drug delivery, elastic fibers/fabrics, wearables, wound healing

## Abstract

Wearables and bioelectronics rely on breathable interface devices with bioaffinity, biocompatibility, and smart functionality for interactions between beings and things and the surrounding environment. Elastic fibers/fabrics with mechanical adaptivity to various deformations and complex substrates, are promising to act as fillers, carriers, substrates, dressings, and scaffolds in the construction of biointerfaces for the human body, skins, organs, and plants, realizing functions such as energy exchange, sensing, perception, augmented virtuality, health monitoring, disease diagnosis, and intervention therapy. This review summarizes and highlights the latest breakthroughs of elastic fibers/fabrics for wearables and bioelectronics, aiming to offer insights into elasticity mechanisms, production methods, and electrical components integration strategies with fibers/fabrics, presenting a profile of elastic fibers/fabrics for energy management, sensors, e‐skins, thermal management, personal protection, wound healing, biosensing, and drug delivery. The trans‐disciplinary application of elastic fibers/fabrics from wearables to biomedicine provides important inspiration for technology transplantation and function integration to adapt different application systems. As a discussion platform, here the main challenges and possible solutions in the field are proposed, hopefully can provide guidance for promoting the development of elastic e‐textiles in consideration of the trade‐off between mechanical/electrical performance, industrial‐scale production, diverse environmental adaptivity, and multiscenario on‐spot applications.

## Graphical abstract

Elastic fiber/fabric as dynamic matrix/carrier for diverse conductor integration, constructing wearables and bioelectronics to bridge organisms (skin, organs, plants, etc.), objects and their around environment, can realize highly safe and efficient interactions. In terms of mechanism, fabrication, and performance, this article presents a profile of elastic fiber/fabric for energy/thermal management, e‐skins, personalized protection/healthcare, disease diagnosis, and intervention therapy.

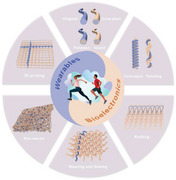

## 1 Introduction

Wearables and bioelectronics play significant roles in constructing soft intelligent interfaces for bridging organisms (such as skin, tissues, organs, plants, etc.), objects, and their surrounding environment, realizing more safe, efficient, and dynamic interactions or energy/information interchange.^[^ ^1^, ^2^, ^3^, ^4^, ^5^, ^6^, ^7^, ^8^ ^]^ Almost all the organisms, such as human beings, animals, and plants, require normal respiration to maintain their respective physiological function and normal growth.^[^ ^9^, ^10^, ^11^, ^12^ ^]^ Functional electronics attached, worn on the organisms’ surface or implanted inside are desired to possess excellent permeability for air/moisture, ensuring comfortability, biosafety, and physiological metabolism.^[^ ^13^, ^14^, ^15^, ^16^, ^17^, ^18^ ^]^ Fibers/fabrics provide excellent bioaffinity, breathability, and mechanical qualities for constructing ideal wearables and biointerface electronics.^[^ ^19^, ^20^, ^21^, ^22^ ^]^ Most existing organisms have uneven surfaces with hard or soft features, suggesting that seamless integration and highly efficient interaction with these multifeature organisms require the devices with excellent mechanical compliance. Elasticity represents the property of a material to adapt mechanical bending, stretching, twisting, and shearing deformations, which can self‐recover to their initial shape or performance after release of the mechanical force.^[^ ^23^, ^24^ ^]^ Therefore, realizing fibers/fabrics with excellent elasticity and electrical function is important for building wearables and bioelectronics, catering for the application requirements of intelligent biointerfaces.

Various fabrication technologies have been relatively mature in exploiting different fiber/fabric‐based electronic devices, such as energy generators, energy storage devices, sensors, actuators, and communicators, and tremendous efforts are devoted to improving the device performances, including power output, power density, sensitivity, responsiveness, actuation force, and communication validity.^[^ ^25^, ^26^ ^]^ Most of these electrical properties rely on the electrode reliability in the devices, for example, to adapt to frequent operation, mechanical deformations, and environmental impacts. Effective electrode integration with fibers and fabrics is one of the ubiquitous and crucial problems in developing various electronic devices. Especially for elastic fibers and fabrics that possess large deformability and relatively low surface energy, the effective incorporation of diverse conductive materials as electrode to construct reliable functional devices is conceivably a considerable challenge. Challenges are primarily remained on the problems such as Young's modulus mismatch, surface roughness adaption, surface wettability tuning, and interface adhesion enhancement.^[^ ^27^, ^28^ ^]^

Fibers with a delicate feel and high aspect ratio have been invented and used for thousands of years. Representative such as cotton, linen, silk, and wool are widely used yarns or textiles for warmth and adornment through conventional processing technologies, including twisting, stitching, knitting, and weaving.^[^ ^25^, ^29^ ^]^ The fast development of synthetic fibers (polyurethane, polypropylene, polyester, etc.) was spurred by people's desire for functional textiles in the twentieth century, and was accompanied by a slew of new production processes such as microfluidic spinning, thermal drawing, printing, and so on.^[^ ^30^, ^31^, ^32^ ^]^ The rapid development of new materials and emerging technologies allow the realization of elastic fibers/fabrics via strategies such as materials innovation, components integration, structure design, and weaving patterning. Accordingly, conductive materials acted as device electrodes could be introduced in fibers with parallel structure, core–shell, and multiaxial structures, in yarns with twisted/coiled structure, and incorporated into fabrics through sewing, weaving, or nonwoven fabrication.^[^ ^30^, ^33^ ^]^ Realizing seamless integration of conductive materials with elastic fibers/fabrics can construct abundant interface electronics, promising to achieve comprehensive interactions of organisms with the environment around, such as energy exchange, perception augmenting, and information communication.

We highlight current breakthroughs in elastic fibers/fabrics for wearables and bioelectronics in this article. First, we systematically introduce the elasticity mechanism, manufacturing methods, and performance of elastic fibers and fabrics, including natural and synthetic materials. Second, we highlight the integration strategies of commonly used conductors with elastic fibers/fabrics, indicating the main challenges and solutions for improving the integration efficiency and enhancing the mechanical/electrical stability of the devices. After that, we emphasize the wearable and biointerface applications of elastic fibers/fabrics derived devices for energy management, sensors, e‐skins, thermal management, personal protection, wound healing, and biosensing and drug delivery. In summary, this article concentrates on elastic fibers/fabrics for precise electrode integration and device development, and highlight the diversified structural design with different geometrics (fiber–yarn–fabric), indicating the advantages and challenges of these devices in mechanical, electrical, and biological applications. It aims to provide insights to encourage the development of elastic e‐textiles and call for more attention to consideration of the performance balance between high electrical performance, industrial‐scale production, diverse environmental adaptivity, and multiscenario on‐spot application.^[^ ^34^, ^35^ ^]^

## 2 Mechanism and Manufacturing of Elastic Fibers/Fabrics

Elastic fiber/fabric means that the material is intrinsically deformable and recoverable (bending, stretching, twisting, shearing) to external stimuli or adaptable to accommodate and maintain the deformations for the requirements in terms of device configuration or application scenarios.^[^ ^36^, ^37^ ^]^ Elastic fibers/fabrics commonly originate from natural and synthetic materials. Natural elastic fibers benefit from the intrinsic deformable microstructure afforded by the physiological features of plants or animals.^[^ ^38^ ^]^ Synthetic elastic fibers/fabrics usually rely on the deformable matrix with dynamic molecule displacement and reassembly, which could alleviate the damage of conductive fillers/paths upon deformation. Herein, we summarized the typical manufacturing methods of elastic fibers/fabrics that could pave the way for related elastic wearables and bioelectronics.

**Figure 1:**
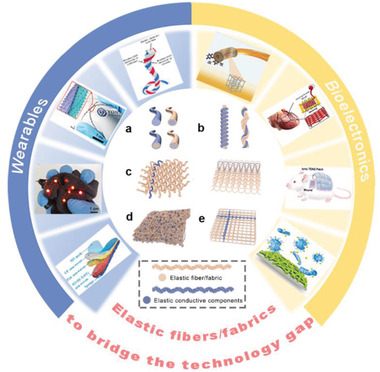
Schematic illustration of the structures of elastic materials in the form of fiber, yarn, fabric, and their integration strategies with electrodes. a) The typical cross‐section structures (core–shell, island structure, etc.) of elastic fibers or elastic conductive fibers. b) Coiling or twisting to realize elastic conductive yarns. c) Accurate incorporation of elastic conductive yarn interspersed in (left) woven and (right) knitted fabrics. d) Diverse incorporation of conductive elastic fibers in a nonwoven fabric via electrospinning, microfluidic spinning, or blowing spinning, etc. e) Accurate printing of elastic conductive ink in 3D elastic fabric.^[^ ^39^, ^40^, ^41^, ^42^, ^43^, ^44^, ^45^, ^46^ ^]^ The surrounding elements are reproduced with permissions from references in the order of counterclockwise on the left half and clockwise on the right one. Reproduced with permission.^[^ ^39^ ^]^ Copyright 2016, American Chemical Society. Reproduced with permission.^[^ ^40^ ^]^ Copyright 2019, Wiley‐VCH. Reproduced with permission.^[^ ^41^ ^]^ Copyright 2020, Wiley‐VCH. Reproduced with permission.^[^ ^42^ ^]^ Copyright 2021, Wiley‐VCH. Reproduced with permission.^[^ ^43^ ^]^ Copyright 2016, Wiley‐VCH. Reproduced with permission.^[^ ^44^ ^]^ Copyright 2017, American Chemical Society. Reproduced with permission.^[^ ^45^ ^]^ Copyright 2021, Elsevier. Reproduced with permission.^[^ ^46^ ^]^ Copyright 2018, The American Association for the Advancement of Science (AAAS).

Wool and silk are famous for their natural elasticity and high modulus due to their intrinsic coil structure or *β*‐phase crystalline with the high alignment of molecule chain.^[^ ^47^, ^48^ ^]^ Due to the natural features and structure stability, the fibers usually are hard to be endowed with higher elasticity. Driven by modern processing technologies, traditional spinning technologies have undergone innovation, where the available materials are no longer confined to naturally elastic wool and silk but further extend to polymers,^[^ ^49^, ^50^ ^]^ semiconductors,^[^ ^32^ ^]^ and conductors.^[^ ^51^ ^]^ The commonly used method to endow stretchability to fiber/fabric is to spin elastic materials directly into fibers or to utilize them as a flexible matrix of other functional fillers for spinning. Elastic polymers are often composed of a dynamic combination of flexible long and stiff, short chains. The prepared fibers are soft in the nonstretched condition due to the random bending of the flexible segments. The external force causes the rigid short‐chain segments to be partially disrupted by conquering intermolecular forces, enabling axial displacement of the amorphous segment chains that induce elongation of the flexible long‐chain segments, resulting in an axial geometric change of the fibers, which appears as macroscopic deformation. When the external stresses are eliminated, the flexible amorphous chain segments could recover from relaxing the fiber.^[^ ^36^ ^]^ Typically, polyurethane (PU) is the most often utilized commercial elastic polymer as it comprises long flexible polyethylene glycol chains and hard moieties that can be stretched up to 500 times their original size.^[^ ^52^, ^53^, ^54^ ^]^ Acted as an elastic matrix, PU has been widely used for functional elastic fibers/fabrics processing by traditional spinning or production techniques, such as wet spinning, thermal drawing, extruding, casting, coating, template, printing, and so on, which allow incorporation of diverse functional components to enrich the function and application of the elastic fibers/fabrics. **Figure** **1**a depicts several typical fiber architectures, such as monofilament, core–shell, and sea‐island.

In addition to the intrinsic deformation effect imparted by the chemical structure of the elastic component, various woven designs of yarn/fabric could also provide elasticity for fabrics/textiles.^[^ ^55^, ^56^ ^]^ Yarns are aggregates of fibers created by twisting or wrapping; their mechanical capabilities are dependent on the arrangement of the fibers in the yarn as well as the properties of the fibers themselves (Figure 1b). The yarn can be torqued by twisting or constructing a core–spun structure, which provokes the structure of the polymer chain segments to become spirally oriented and the fibers in the yarn to become more compact, a process that is also considered necessary to give the yarn a certain strong elongation and a stable appearance.^[^ ^57^, ^58^ ^]^ Yarns with tight and durable structures have strong elasticity, whereas those with loose forms have relatively poor elasticity. The performance of the composite yarn is also related to the composition and content of the fibers in the blend. The addition of stretch yarn blends also improves the overall elasticity and recovery of the yarn.

Figure 1c shows the possible ways that the elastic substance/elastic yarn can be combined with the fabric. Fabrics are soft items with certain mechanical qualities and thickness formed from textile fibers or yarns.^[^ ^59^ ^]^ Different textiles can be classified as woven, knitted, or nonwoven fabrics‐based on their respective processing technique. Fabric elasticity, like yarn elasticity, is primarily determined by material selection, yarn structure, density, and fabric structure and density. How elastic yarns are introduced into knitted and woven textiles is similar, the following is an example of knitted fabrics. The stitches of a knitted fabric can deform significantly due to the curved shape of the stitches and the fact that yarns can cross from one thread into a neighboring stitch.^[^ ^60^, ^61^ ^]^ Knitted textiles have excellent elongation capabilities, owing to their high knitting strength and less mutual extrusion and friction between the threads, in which the loops have a large deformation region during stretching, and the yarns would move in dislocation at the interlacing point. Furthermore, the sensible selection of yarn is critical in the weaving process. For example, during the interlacing and interlocking of electrode yarns, weaving and knitting operations often generate high tensile and tear resistance, as can intersperse stretch yarns.^[^ ^62^ ^]^

Another available method is to incorporate the conductive fillers as a random insertion in the fiber materials by electrospinning, microfluidic spinning, and blowing spinning (Figure 1d), or orderly assemble active electrodes by 3D printing technology (Figure 1e). They could provide versatile structure and function for e‐textiles; the processes highly rely on versatile, functional inks with adaptivity for accurate printing or large‐scale fabrication.^[^ ^63^, ^64^ ^]^ Integration of the traditional textile engineering processes and emerging micro/nanomachining technologies could realize elastic fibers/fabrics with advanced performances, providing important substrates and materials for further constructing fiber/fabric‐based elastic electronic devices with mechanical compliance and interface adaptivity, which is promising to bridge the technology gap between wearables and bioelectronics.

## 3 Integration Strategies of the Electrode with Elastic Fibers/Fabrics

The electrode is one of the crucial components of electronic devices for charge transfer, current collecting, and signal transducing, ensuring the devices could normally function in the fields such as energy harvesting, energy storage, sensing, actuating, transducing, and communicating.^[^ ^65^, ^66^ ^]^ For elastic fibers/fabrics‐based devices, the seamless incorporation of deformable electrodes remains a huge concern due to their mismatch of mechanical performances and surface properties. Various functional conductors have been developed for function integration to realize devices with electrode in form of fiber, yarn, and textile, such as carbon‐based materials,^[^ ^67^, ^68^, ^69^, ^70^ ^]^ nanometals,^[^ ^69^ ^]^ 2D transition metal carbides/nitrides (MXene),^[^ ^69^ ^]^ metallogels (MOG),^[^ ^71^, ^72^ ^]^ liquid metals (LM),^[^ ^73^, ^74^ ^]^ liquid crystals,^[^ ^73^, ^75^ ^]^ and so on. In the following section, we briefly summarized the typical works to indicate the crucial techniques for integration of different conductors with fibers/fabrics, realizing various function devices.

### 3.1 Elastic Conductive Fibers

**Figure 2:**
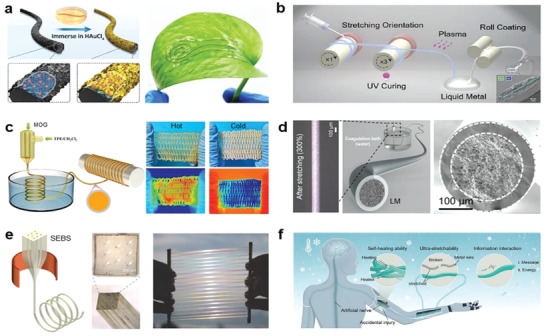
Integration strategies of conductors with the elastic fibers. a) A stretchable and conductive fiber acts as electrochemical sensor attached on a leaf for H_2_O_2_ detection. Reproduced with permission.^[^ ^69^ ^]^ Copyright 2022, Elsevier. b) Schematic fabrication of high‐contractile electrothermal‐driven liquid crystal elastomer fibers. Reproduced with permission.^[^ ^73^ ^]^ Copyright 2021, Wiley‐VCH. c) Stretching fabrics woven from metalloges/TPE ionic conducting fibers with wide temperature adaptivity. Reproduced with permission.^[^ ^76^ ^]^ Copyright 2022, American Chemical Society. d) Production schematic and state under large deformation of LM sheath–core microfibers. Reproduced with permission.^[^ ^77^ ^]^ Copyright 2021, The American Association for the Advancement of Science (AAAS). e) Thermally drawn SEBS fibers with multichannels to encapsulate liquid metals. Reproduced with permission.^[^ ^74^ ^]^ Copyright 2018, Wiley‐VCH. f) Ultrastretching and self‐healing ionic hydrogel‐based artificial nerve fiber with cryogenic environments tolerance. Reproduced with permission.^[^ ^78^ ^]^ Copyright 2022, Wiley‐VCH.

Fiber is the basic unit of yarns/fabrics, elastic conductive fiber could help to construct individual fiber device as well as to realize accurate processing of elastic yarns/fabrics devices.^[^ ^30^, ^79^, ^80^ ^]^ There are versatile strategies to realize elastic conductive fiber, such as coating, cospinning, coaxial spinning, thermally drawing and solution extrusion. Wetting spinning is commonly used for composite functional fiber processing, such as the blended precursor of elastomer matrix and conductive fillers could produce elastic conductive fiber.^[^ ^81^, ^82^, ^83^ ^]^ For example, thermoplastic PU mixed with multiwalled carbon nanotubes (CNTs) was applied to produce an elastic fiber with adjustable electrical conductivity, good elasticity of 310% and high tensile strength of 28 MPa were demonstrated.^[^ ^68^ ^]^ Similarly, based on the catalytic capabilities of gold nanostructures (Au CNs), Lan et al. designed conductive and stretchable fiber sensors with electrochemical properties for detecting H_2_O_2_.^[^ ^69^ ^]^ As illustrated in **Figure** **2**a, the resultant fibers were made up of MXene and CNTs embedded in PU with cobblestone‐like Au CNs on their surfaces. The fiber possessed exceptional electrical conductivity and mechanical compliance, as well as good conformality on curved leaves, allowing for fast H_2_O_2_ reduction at an applied potential of 0.1 V. As we know, due to the relative fine cross‐sectional area of fibers, the conductive fillers in the composite fiber are hard to form reliable conducting path upon deformation, thus the fibers are commonly limited in a trade‐off between conductivity and stretchability. This kind of elastic fibers usually behaves as a high conductive response to mechanical deformations, more suitable to act as resistive sensors rather than electrodes.

It inspires that the continuous conductive structure maintained upon deformation is important for realizing reliable conductivity. More efforts were dedicated to developing elastic fibers with various topographical structures, such as core–shell fibers that allow the continuous electrode to form as core or shell of the fibers, capable of improving the conductive stability. Through the coating process of graphite sheet, Zhang et al. developed a silk fiber based strain sensor. After 3000 cycles of stretching within 15% strain, the sensor retained good sensitivity with a gauge factor of 14.5.^[^ ^67^ ^]^ Under electrical stimulation, Sun et al. created electrothermally sensitive liquid metal containing liquid crystal elastomer (LM‐LCE) fibers with rapid contraction rates and high contraction ratios. Figure 2b depicts the formation of a single fiber by decorating liquid metal on a liquid crystal fiber, followed by lamination of two LM‐LCE fibers to generate an LM‐LCE elastic conductive fiber with cross‐section akin to a core–shell structure. The LM‐LCE fiber had a maximal contraction rate of 284% s^−1^ and a contraction ratio greater than 40%. Under the dual reaction of electricity and heat, this design may imitate artificial muscles and perform autonomous power actuation.^[^ ^73^ ^]^ In another example, Wang et al. succeeded in incorporating twist into LCE fibers, i.e., the orientation of the fibers was tilted, which enabled LCE fibers not only to complete the shrinkage in the longitudinal length under thermal stimulation, but also to be accompanied by a high degree of torsion and deformation.^[^ ^75^ ^]^ The elastic LCE fiber exhibits great potential for actuators and soft robotics.

Coaxial spinning is a more efficient means to prepare stretchable conductive fibers with core–sheath structure, wherein the elastomer matrix or deformable conductive materials usually act as shell to encapsulate the conductive fillers and enables conductivity of the fiber not highly compromised during stretching.^[^ ^84^, ^85^ ^]^ For example, coaxial wet spinning was utilized to manufacture elastic fibers with MXene/PU sheaths and pure PU cores that can withstand tensile strain of up to 150% for 1000 cycles and maintain good stability.^[^ ^83^ ^]^ MOG with weak noncovalent interconnections can sustain reversible sol–gel transitions in response to external stimulus such as vibration, light, pH, and temperature.^[^ ^71^, ^72^ ^]^ As shown in Figure 2c, MOG acted as a conductive core, a core–shell elastic fiber of MOG–thermoplastic elastomer (TPE) was developed for wearable sensors. The fiber showed fast response capability (100 ms) for sol–gel transition and stable electrical conductivity even under large deformation. Good stretchability of ≈100% and cycling stability (>3000 cycles) were demonstrated at temperature ranges from −50 to 50 °C. Application potential of the fiber was presented by attaching to the elbows, wrists, fingers, and knees for human motions monitoring under extreme conditions.^[^ ^76^ ^]^ Figure 2d shows a highly conductive superelastic fiber developed on the basis of a core–shell structure. The inner core, consisting of LM and fluoroelastomer (the same elastomeric material also presents as a shell), exhibited codeformation with the help of dipole moment force when being stretched, achieving a small change in resistance of only 4% at a large deformation of 200%, showing great promise for smart fabrics and self‐powered sensors.^[^ ^77^ ^]^

Important progress of elastic conductive fibers was achieved via exploitation of various manufacturing processes such as coating decoration, cospinning, and coaxial spinning.^[^ ^86^, ^87^ ^]^ However, the fibers with relatively simple configurations would be inadequate to meet the increasing requirements for complex application, such as smart fibers for intelligent optoelectronics and biomedical applications.^[^ ^88^, ^89^ ^]^ Thermal drawing technology was originally used to make optical fibers, which allows diverse functional materials to be synchronously configurated into one fiber via different channels, had evolved into a potent tool for the manufacture of multifunctional elastic fiber electronics.^[^ ^90^, ^91^, ^92^ ^]^ Typically, to achieve fibers with high elasticity and conductive stability, liquid metal was commonly used as a conductive substance in the holes (as the core) due to its excellent conductivity and deformability. As shown in Figure 2e, a nozzle with multiholes was applied for thermal drawing of poly(styrene‐*b*‐(ethylene‐*co*‐butylene)‐*b*‐styrene) (SEBS), the liquid metal fed initially could flow freely through the microchannels during the subsequent thermal drawing process since the volume stays constant, resulting in stretchable SEBS fiber with multiconductive paths and tens of kilometers in length.^[^ ^74^ ^]^ Furthermore, the thermal drawing procedure is also competent in manufacturing hollow superelastic fibers. As shown in Figure 2e, the poly(vinyl alcohol) (PVA) pellets were first molded into rods of various geometrics such as cylindrical, triangular, rectangular, etc., which acted as templates to be then tightly wrapped by the SEBS film to form the preform. After soaking in hot water to remove the PVA template, hollow SEBS elastic fibers with various shapes of cross‐section were obtained.^[^ ^93^ ^]^ Therefore, thermal drawing process could effectively enrich the structure and function of elastic conductive fiber; it provides expanded options for creating varied electronic fibers to meet different application requirements.

Considering the requirement of mass production of functional fibers, solution extrusion has been proved a suitable technique for high‐speed mass manufacturing of elastic conductive fibers due to the moderate process conditions.^[^ ^70^, ^94^ ^]^ The pioneering works were demonstrated by Peng's group for mass production of batteries, displays, and transistors.^[^ ^27^, ^95^ ^]^ Typically, they designed a three‐channel spinneret in which fiber cells with parallel cathodes and anodes coated with chitosan gel electrolyte ink composed of polyvinyl alcohol and lithium sulfate (Li_2_SO_4_) were continually extruded, stretched, and collected at high speed (≈250 m h^−1^).^[^ ^96^ ^]^ Since this preparation process did not need demanding manufacturing conditions, it could be universally utilized for the quick mass manufacture of a wide range of other electronic devices, such as solar cells, supercapacitors, and light‐emitting electronic displays.^[^ ^97^, ^98^, ^99^ ^]^ For example, fiber cells can be used in conjunction with fiber sensors and electroluminescent fibers to build body monitoring micronetworks. The lightweight fiber cells are programmed into the textile tissue to provide energy to support health signals to the textile display without compromising user comfort.^[^ ^100^ ^]^ This innovative strategy opens up new possibilities for developing fiber‐based self‐powered electronics. For example, a multifunctional ionic hydrogel fiber with rapid self‐healing, superstretching capability, and sustained electrical conductivity was demonstrated for simulated artificial nerve fibers (Figure 2f).^[^ ^78^ ^]^ In addition to quick signaling responses, the produced ionic hydrogel fibers can achieve rapid self‐healing within 10 min and tensile deformation of up to 7000%. More interesting, these performances were well preserved even in extremely cold temperatures (−78.5 °C) with 200% repeated stretch/release 10 000 cycles, indicating a promising potential for intelligent robots to complete tasks independently under harsh circumstances. Similarly, Chen and co‐workers devised hydrogels that accurately adjust the pH of the wound surface and speed up wound healing. This concept could be realized by microfluidic assembly technology, forming macroscopically structured populations of fibers, planes, and 3D gels to adapt to specific wound surface morphologies. Self‐regulation of the pH value of the affected area can enhance the proliferation, migration, and activity of new cells, thereby greatly accelerating the healing of chronic wounds. This work presents a promising strategy to design wound healing materials with diverse architectures and great potential for chronic wound management.^[^ ^101^ ^]^

### 3.2 Elastic Conductive Yarns/Fabrics

Yarns and fabrics with higher mechanical strength and larger areas are the more commonly used forms in textile engineering processing and in our daily life, they act as substrates that could provide abundant designs and functions for electronics.^[^ ^30^, ^102^ ^]^ The comfort, mechanical properties and function potential of fabrics/garments are primarily determined by the material, arrangement, and the integrated structure of yarns.^[^ ^103^ ^]^ Incorporating conductive materials into the yarn/fabric may affect the original textile properties such as permeability, affinity, and comfortability, posing a significant challenge for developing comfortable wearable electronics.^[^ ^27^, ^104^ ^]^ Tremendous efforts were being done to seek effective ways of merging conductivity, stretchability, comfort, breathability, and responsive functionality. A relatively acceptable way is patterning the prefabricated elastic conductive yarns or printable elastic conductive inks to construct electrodes for device configuration.

**Figure 3:**
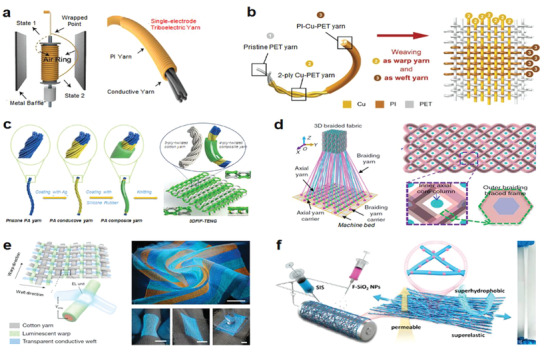
Integration strategies of conductors with the elastic yarns/fabrics. a) Triboelectric yarns with flame retardancy for precise rescue location, real‐time route guidance, and noise reduction. Reproduced with permission.^[^ ^108^ ^]^ Copyright 2020, Wiley‐VCH. b) Woven washable TENG with plain structure. Reproduced with permission.^[^ ^109^ ^]^ Copyright 2016, Wiley‐VCH. c) Core–sheath yarns based TENG with 3D double‐faced interlock knitted structure for multifunctional wearable devices. Reproduced with permission.^[^ ^110^ ^]^ Copyright 2020, Elsevier. d) Fabric‐TENG fabricated by 3D four‐step rectangular braiding technology. Reproduced with permission.^[^ ^111^ ^]^ Copyright 2020, Springer Nature. e) EL arrays interconnected by luminescent warp and transparent conductive weft. Reproduced with permission.^[^ ^98^ ^]^ Copyright 2021, Springer Nature. f) SPSM enabled by synchronous electrospinning and electrospraying. Reproduced with permission.^[^ ^92^ ^]^ Copyright 2022, Elsevier.

For a yarn‐based device, the conductive components can act as the core or shell of the yarns to serve as a functional electrode. For example, Zhang and co‐workers prepared yarns with a core–spun yarn structure by wrapping nanosilk fiber films around rotating CNTs. The produced yarns showed excellent electrical conductivity, strong mechanical strength, and great comfort due to the high mechanical softness and the biocompatible silk skin.^[^ ^105^ ^]^ Wang and co‐workers designed a single electrode triboelectric nanogenerator (TENG) with a core–spun yarn structure composed of flame‐retardant polyimide (PI) twisted conductive yarn (**Figure** **3**a). The conductive yarn was entirely covered by the outer flame‐retardant PI yarn, which produced a triboelectric active layer and gave the composite yarn exceptional flame retardant qualities. This flame retardant TENG can generate stable triboelectric output via the interaction between the conductive core and PI shell, which was demonstrated as an effective instrument for fire rescue. Operating in a single electrode mode to harvest energy from skin or an object (another electrode), the yarn could act as a self‐powered sensor to pinpoint the location of survivors and assist in timely victim search and rescue.^[^ ^106^ ^]^ In addition, elastic conductive yarns were also designed by twisting the conductive film alone or in combination with other yarns. For example, spring‐like CNT films, were utilized as superelastic yarn strain sensors following torsion, with exceptional tensile characteristics up to 285% of their own.^[^ ^107^ ^]^

Moreover, unlike the traditional longitudinal elastic stretching/contraction, the ability to reversible fusion and fission fibers within a yarn in response to external stimuli is also an interesting form of elastic yarn. A unique example was demonstrated by Gao and co‐workers, who discovered that wet spun graphene oxide (GO) yarns (a collection of densely packed individual fibers) deliver reversible fusion and fission behaviors under solution induction.^[^ ^112^ ^]^ When immersed in water, the GO yarn swelled and finally broke into multiple separate fibers; away from water, the individual fibers shrank in volume and gradually aggregated toward the center, fusing back into the original densely packed aggregate state upon drying. This structural conversion and response drive satisfied the requirement for GO‐based elastic yarns to be universally usable in several application situations, such as reversible conversion between 3D rigid rods and 2D flexible webs and provide actuation for release and recycling of functional components or target objects.

For electronic fabrics/textiles, the conductive yarns acted as functional electrode with different patterns and spatial distribution could be incorporated by changing the textile structure, realizing diverse devices. Using the stronger contact effect at yarns junctions that could simulate the stacking structure of functional layers, different devices could be achieved on a large‐scale through the conventional textile engineering technologies. TENG is g typical device that relies on the interface contact to produce electricity. For example, Zheng and co‐workers utilized Cu‐coated polyethylene terephthalate (Cu‐PET) as the warp yarn and polyimide (PI)‐coated Cu‐PET (PI‐Cu‐PET) as the weft yarn to weave the metal yarns into a plain fabric TENG (Figure 3b),^[^ ^109^ ^]^ which consisted of a lot of TENG units at the yarns junctions, the maximum output current density reached up to 15.50 mA m^−2^. It was demonstrated as a self‐powered respiratory monitor to record human respiratory rate and depth.

Multistructure textiles that differ from regular fabrics have achieved numerous new advances in fabric structure, such as 3D structure fabrics, due to the continual change and development of fabric processing technologies.^[^ ^113^, ^114^ ^]^ 3D fabrics commonly include 3D woven fabrics, 3D knitted fabrics and 3D nonwoven fabrics, which have an expanded thickness direction in addition to the 2D structure of planar materials. The fabrics with 3D structures deliver enhanced capability to accommodate multidirectional responsiveness and larger deformation, promoting the device performance. For example, a 3D double‐sided interlocking fabric TENG was demonstrated by knitting cotton yarn and polyamide (PA) composite yarn with silver‐plated four‐ply twisted PA conductive yarn through double needle bed flat knitting technology, shown in Figure 3c.^[^ ^110^ ^]^ This fabric‐based TENG with intrinsic 3D elastic structure can self‐response to nearly arbitrary complex deformations (such as lateral, longitudinal) and generate triboelectric outputs via the contact–separation between the adjacent yarns, demonstrating good potential for self‐powered wearable devices. Besides, Wang and co‐workers developed a 3D five‐way weave structured TENG with the spatial frame column between the outer woven yarn and the inner axial yarn, which had high pressure response resilience, high power output capacity, high pressure sensitivity, and vibration energy collecting ability (Figure 3d).^[^ ^111^ ^]^ The works present important potential of 3D fabric for self‐powered devices, wearables, and human–machine interfaces.

Besides TENG, some other devices had also been realized by virtue of the contact functions of yarns junctions in fabric. For instance, Peng and co‐workers reported a 6‐m‐long and 25‐cm‐wide electronic display textile based on the premise that active sites could be generated by cross‐contact of warp and weft yarns. They wove the conductive weft yarn generated by melt spinning with PU gel as the skin layer (light transmittance more than 90%) and the luminous warp yarn prepared by depositing commercial ZnS phosphor on the silver‐plated conductive yarn. As shown in Figure 3e, micrometer‐scale electroluminescence (EL) units can be generated when the conductive weft yarns and the luminous warp yarns come into touch under an electric field.^[^ ^98^ ^]^ The EL units maintained stability even when bent, stretched or pressed thanks to good resistance of PU. Accordingly, large‐scale functional devices including fabric‐based batteries and transistors had also been demonstrated.^[^ ^100^, ^115^ ^]^ The traditional textile technology embraced with new functional materials and device types promises attractive potential to push the commercialization of electronic textiles for future intelligent wearables and human–machine interfaces.

In addition to the conventional woven elastic fabrics, elastic nonwovens also play vital role to enrich the device configuration and function.^[^ ^116^, ^117^, ^118^, ^119^ ^]^ To tackle the main shortcomings of the most traditional nonwoven materials that have low deformability or poor elasticity due to the labile fibers network stacked physically, Li et al. proposed a series of strategies to develop micro‐/nanofiber nonwovens with interlocked structure, promoting the stretchability and structural stability. For example, They developed an electrospinning polyvinyl fluoride‐hexafluoropropylene (PVDF‐HFP) nonwovens embedded with electrostatically sprayed styrene–ethylene–butylene–styrene (SEBS) microspheres, where the distributed SEBS elastic microspheres acted as a binder can effectively fix the nanofibers, so that the fibers network showed good reversibility to accommodate the stretching deformation.^[^ ^120^ ^]^ By screening printing SEBS/LM/silver flakes (Ag FKs) elastic conductive ink on the nonwoven fabric, they demonstrated a single electrode TENG with good stretchability (≈120%) and breathability for self‐powered sensing. Recently, Li et al. further proposed a co‐solvent welding strategy for in situ generation of interlocked fiber junctions during electrospinning, which can significantly improve the stretchability of nonwoven fabrics.^[^ ^92^ ^]^ By synchronous electrospinning of styrene‐isoprene (SIS) block copolymers and electrospraying of the fluorinated titanium dioxide (F‐SiO_2_) nanoparticles, they demonstrate an SIS microfiber membrane with superelasticity (≈3600% tensile strain), good air/moisture permeability (90% of cotton) and superhydrophobicity (water contact angle 154.2°) (SPSM) (Figure 3f). Moreover, they achieved robust integration of SPSM with different elastic conductors (sputtered gold nanolayer, filtrated LM/Ag NWs and printed SIS/LM/Ag FKs) by means of cosolvent etching effect. The permeable superelastic fibers‐based conductors demonstrated optional mechanical/electrical stability and were applied as wearable and on‐skin TENGs for long‐term usage.

### 3.3 Printable Inks for Elastic Conductive Fibers/Fabrics

The aforementioned integration methods of electrode in different fibers/yarns/fabrics indicated respective advantages of the traditional textile process and the emerging post‐incorporation technology. The processes to elastic conductive coaxial fibers, twisted yarns, and woven fabrics could realize accurate device structure and large‐scale manufacturing. Apart from this, the direct integration of conductive materials with existing fabrics attracted increasing attention since it inherits the merits of post‐treatment technology of textile that allows efficient transformation from ordinary textiles to functional e‐textiles.^[^ ^121^, ^122^, ^123^ ^]^

Printing represents a class of technology that can achieve high throughput, high resolution, and patterning fabrication of functional materials on different substrates. Inks with different rheological properties to meet the diverse interface properties of substrates is a fundamental concern for a diverse range of printing techniques. Functional ink with advantages in mechanical, electrical, and interface features represents a promising direction for wearables and bioelectronics exploitation.^[^ ^124^, ^125^, ^126^ ^]^ Elastic conductive ink promises significant potential to act as functional electrode for fabrics/textiles. However, the complicated structure, high porosity and surface roughness of fabrics would increase the integration difficulty with inks as well as due to their unmatched Young's modulus and interface properties.^[^ ^127^, ^128^ ^]^ Increasing efforts were devoted to developing highly elastic conductive inks with stable electricity to dynamically accommodate substrates’ deformation. In general, elastic conductive inks consist of elastomer matrix and conductive fillers, which determine the deformability and conductivity. The elastomers commonly include polydimethylsiloxan (PDMS), PU, ethylene‐vinyl acetate (EVA), SEBS, and SIS. Conductive fillers such as carbon black, graphite, graphene, CNT, MXene, Ag NPs, Ag NWs, Ag FKs, and LM are often used for ink preparation.

**Figure 4:**
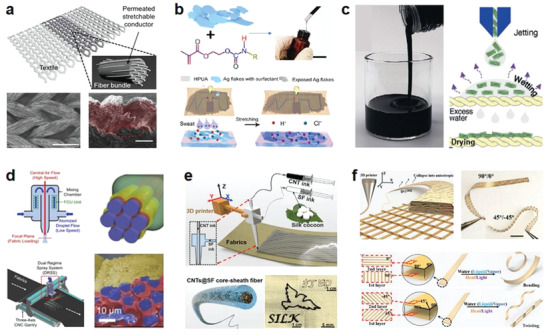
Printable inks for direct integration into fabrics. a) A fluoroelastomer‐Ag FKs elastic ink with high penetration in textile. Reproduced with permission.^[^ ^130^ ^]^ Copyright 2017, Wiley‐VCH. b) A printable HPUA‐Ag FKs elastic ink on textile with enhanced conductivity upon contacting sweat. Reproduced with permission.^[^ ^131^ ^]^ Copyright 2021, The American Association for the Advancement of Science (AAAS). c) An additive‐free MXene ink with good printability on textile. Reproduced with permission.^[^ ^133^ ^]^ Copyright 2020, Wiley‐VCH. d) A dual regime spray technology with improved printing accuracy on textiles. Reproduced with permission.^[^ ^135^ ^]^ Copyright 2022, Wiley‐VCH. e) 3D printed coaxial CNTs‐SF fibers on textiles for facile construction of e‐textile. Reproduced with permission.^[^ ^139^ ^]^ Copyright 2019, Elsevier. f) 3D printed GO–SA bilayer strip with multiactuations to various stimuli. Reproduced under the terms of the Creative Commons CC‐BY licensen.^[^ ^140^ ^]^ Copyright 2020, The Authors. Published by Wiley‐VCH.

Screen printing is a typical process that could realize functional patterns by blade coating the ink on a custom‐made stencil. By designing stencils with different geometrics, conductive patterns with difference in pattern and size can be achieved, it is a classic printing technology highly usable for rough substrates such as fabric and textiles.^[^ ^129^ ^]^ To realize conductive ink highly adaptive to the porosity and deformability of textile, Someya and co‐workers developed an Ag FKs‐based elastic ink using fluoroelastomer (poly(vinylidene fluoride‐*co*‐hexafluoropropylene) and a low boiling point solvent of 2‐2(butoxyethoxy)ethyl acetate (or butyl carbitol acetate). High penetration was realized on the ink to infiltrate well and conform to the textile structure, demonstrating a printed fabric with an initial resistance of 0.06 sq^−1^ that could sustain 450% tensile strain and maintain only 70 times resistance increase (**Figure** **4**a).^[^ ^130^ ^]^ In addition, Lee and co‐workers developed a hydrophilic polyurethane acrylate (HPUA) as a matrix of Ag FKs to tune the ink wettability to adapt to the hydrophilic fabrics and sweat transmission. More interesting, they revealed that enhanced conductivity occurred on the ink upon it was in contact with sweat (Figure 4b).^[^ ^131^ ^]^ The enhancement mechanism was verified as that Cl^−^ and lactic acid in sweat accelerated the removal of the insulating surfactant from the Ag FKs, enabling better conductive connections between the exposed Ag FKs and reduced resistance in both the relaxed and stretched states. The proof of concept could provide insights and inspiration for prolonging the life‐time of future printed stretchable electrodes.

Inkjet printing is a relatively more precise deposition technology, allowing accurate pattern of micro‐/nanomaterials.^[^ ^132^ ^]^ Conductive ink with suitable composite size and liquid viscosity is important to ensure a smooth printing process. For example, with no need of other additives, Gogotsi and co‐workers developed a water‐based Ti_3_C_2_T*_x_* MXene ink capable of direct printing by controlling the MXene flake size and ink concentration to regulate its rheology. Once the droplet reaches the substrate, it penetrates and adheres firmly to the substrate due to the compatibility between surface tension of the ink and surface energy of the substrate, the formed ink pattern can dry quickly free of post‐treatment (Figure 4c),^[^ ^133^ ^]^ the printed electrode was applied for micro‐supercapacitors. Recently, Chang et al. reported a dual regime spraying that is an upgraded technology based on conventional inkjet printing, capable of compensating the specification constraints and accuracy deficiencies in mass production.^[^ ^134^ ^]^ As shown in Figure 4d, silver nitrate (AgNO_3_) nanoparticles seamlessly penetrated the fabric through atomized droplets under the action of two airflow modules at high and low speeds, allowing precise customization of the electrode pattern and fabric design while retaining the fabric's inherent comfort, breathability, and mechanical compliance.^[^ ^135^ ^]^ The produced e‐textiles were evaluated for monitoring horses’ dynamic activity and physiological health indications, confirming the viability and universality of this process enhancement. This dual regime spraying effectively improves the applicability of inkjet printing to adapt complex textile substrates.

3D printing is a promising technology for flexible device fabrication due to its accuracy, high efficiency, and extensive material adaptation.^[^ ^136^, ^137^, ^138^ ^]^ Zhang et al. proposed a coaxial filament‐spray 3D printing technology to enrich structure and function of e‐textiles for varied scenarios. For example, by printing fibers with CNTs as a conductive core and silk‐sin protein (SF) as shell, they demonstrated various functional patterns on textiles. Utilizing the triboelectric effect of SF layer, they presented a power textile with good softness and comfort to harvest mechanical energy from human motion (Figure 4e).^[^ ^139^ ^]^ In addition, Zhang et al. printed electronic strips with brick‐and‐mortar architecture built by aligned GO flakes and sodium alginate (SA) matrix (Figure 4f). Based on the rapid responsiveness of GO to humidity stimuli and the difference in the vertically stacked bilayer structure, the resulting strip can be reversibly bent or twisted under water drive by controlling the print paths of the bottom and top layers, that is, by locally regulating the GO orientation.^[^ ^140^ ^]^ To prove this theoretical feasibility, a vapor‐responsive gripper was fabricated to demonstrate cotton balls’ intelligent gripping and releasing behavior in diverse humidity settings, ushering in a new era for developing intelligent responsive textile systems.

Overall, the printing technologies show respective superiorities in printing precision and processing efficiency.^[^ ^141^, ^142^, ^143^, ^144^, ^145^ ^]^ One of the main challenges encountered by all the printing technologies for porous and elastic fabric/textile substrates is the accurate control of electrode patterns to adapt different substrates. The solutions to tackle this concern could be printing parameters regulation, substrate pretreatment, and a well‐designed stencil, which cause tedious fabrication and increase energy consumption. Developing new elastic conductive inks that are universally applicable for different textiles would be a feasible route to conquer the challenge. Considering the properties of conductive ink in good osmosis, low diffusivity, self‐attachability, and high deformability/stretchability is essential for realizing multifunctional ink to adapt the complex substrates with high elasticity and diverse wettability.

In short, various technologies have been demonstrated for flexible electrode integration with elastic fiber, yarn, and textile, where stable elastic electrodes are essential for the devices to adapt large deformations, device configurations, and application scenarios such as with high temperature, high humidity, contaminations, and washing. Typical examples for processing fiber, yarn, and textile into various wearables or bioelectronics are summarized in **Table** **1**, wherein the advantages and challenges of different materials in electrode integration, device fabrication, operation, cost, and performance are systematically compared. Briefly, 1) conductive fiber/yarn is the more suitable electrode for daily fiber/yarn to construct functional devices. Weaving or printing technology is largely adoptable for fabricating electrode and achieving fabric/textile‐based devices. 2) Fiber/yarn presents advantages for sensors, or 1D energy devices that require precise structure design, fabric/textile with larger area is superior in realizing high power density/output. 3) Twisting or knitting is effective to realize structural elasticity of yarns or fabrics. High mechanical/electrical stability of raw materials is important for ensuring the processing and mass production. 4) Interface adaptivity and stability between elastic electrodes and fiber/textile substrates are significant for mechanical/electrical stability of devices. Accurate design of the yarns junctions by traditional textile technology is a promising strategy to simulate device diverse stacking configurations and realize scalable production. 5) Material option and smart encapsulation could be considered for improving device environment adaptivity and stability, such as moisture/temperature resistance, self‐cleaning, skin‐affinity, and biocompatibility.

**Table 1:** Advantages and challenges of elastic fiber, yarn, fabric for diverse applications

Type	Fabrication	Advantages	Challenges	Typical application
Fiber	Wet spinning	Intricate geometrics (cospinning, core–shell, island) Extensive raw materials One‐step fabrication	Inferior uniformity Slow processing Process discontinuity	Sensor^[^ ^68^, ^69^ ^]^ TENG^[^ ^77^, ^81^ ^]^ Battery^[^ ^96^, ^146^ ^]^ Biosensing^[^ ^71^, ^147^ ^]^ Drug delivery^[^ ^148^, ^149^ ^]^
	Thermal drawing	Prefabs diversity Component tunability Accurate integration	Material restrictions Harsh condition Costly equipment	Sensor^[^ ^74^, ^93^ ^]^ Battery^[^ ^74^, ^150^ ^]^ TENG^[^ ^93^, ^151^ ^]^ TEG^[^ ^32^, ^96^ ^]^
	Finishing	Integration diversity Diversified functionality Simple operation, low cost	Sacrifice of fiber merits Poor structure uniformity Inferior washability/ durability	Sensor^[^ ^67^, ^73^ ^]^ Battery^[^ ^152^, ^153^ ^]^
Yarn	Twisting	Structure diversity Realization of elasticity Mass production	Limitation in high elasticity Limitation in device construction Poor performance	Sensor^[^ ^103^, ^154^ ^]^ TENG^[^ ^155^, ^156^ ^]^ PENG^[^ ^157^, ^158^ ^]^ Thermal management^[^ ^159^, ^160^ ^]^
	Finishing	Materials diversity Diversified functionality Simple operation, low cost	Sacrifice of yarn twisting effect Lack of yarn wearable merits Inferior washability/ durability	Sensor^[^ ^161^, ^162^ ^]^ SC^[^ ^163^, ^164^ ^]^ TENG^[^ ^165^, ^166^ ^]^ TEG^[^ ^167^, ^168^ ^]^
Fabric textile	Weaving	Customized device patterns Good mechanical strength Scalability and high performance	Limitation of raw material in mechanical and electrical stability Low structural elasticity Precise fabrication of contact junctions for different devices	Sensor^[^ ^169^, ^170^ ^]^ SC^[^ ^171^, ^172^ ^]^ TENG^[^ ^109^, ^111^ ^]^ PENG^[^ ^173^, ^174^ ^]^ TEG^[^ ^175^, ^176^, ^177^ ^]^
	Knitting	Intrinsic structural elasticity Scalability, high mechanical strength and outputs Easy incorporation of e‐yarn	Limitation of raw material in mechanical and electrical stability Precise fabrication of contact junctions for different devices	Sensor^[^ ^178^, ^179^ ^]^ SC^[^ ^180^, ^181^ ^]^ TENG^[^ ^111^, ^182^ ^]^ TEG^[^ ^175^, ^183^ ^]^
	Nonwoven	Components incorporation Diversity in membrane, yarns/fabrics Mass production	Limitation in single fibers with controllable properties Poor mechanical properties Challenge in diverse process	TENG^[^ ^92^, ^184^ ^]^ PENG^[^ ^185^, ^186^ ^]^ Personal protection^[^ ^46^, ^187^ ^]^
	Printing	Versatile printing methods High precise patterning High throughput	Complex process parameters Costly equipment Limitation in new materials	SC^[^ ^188^, ^189^ ^]^ Battery^[^ ^190^, ^191^ ^]^ TENG^[^ ^192^, ^193^ ^]^ PENG^[^ ^194^, ^195^ ^]^
	Finishing	Technology diversity Extensive material adoption Large scale functionality	Low fabrication accuracy Sacrifice of textile wearability Inferior washability and durability	Sensor^[^ ^196^, ^197^ ^]^ SC^[^ ^198^, ^199^ ^]^ Battery^[^ ^200^, ^201^ ^]^ TENG^[^ ^202^, ^203^ ^]^ Thermal management^[^ ^204^, ^205^ ^]^

## 4 Elastic Fibers/Fabrics for Wearables

Wearable technology is described as a networked hybrid device that could be worn on or within the body and incorporated into the user's everyday life and motions. With the development of advanced materials and technologies, conventional textiles (fibers, yarns, and fabrics) with skin‐affinity and permeability superiorities have been endowed with new vitality in wearables.^[^ ^206^ ^]^ Being one of the ideal mediums, textiles are expected to act as a bridge to promote the development of wearables, biointerfaces, and human–machine–environment interactions.^[^ ^25^, ^70^, ^206^ ^]^ Comprehensively considering the demands of practical applications, performances such as mechanical compliance, self‐attachability, high fidelity, and comfort are desired on the wearables.^[^ ^194^, ^207^, ^208^ ^]^ Devices based on elastic fibers/fabrics shows fascinating advantages in meeting high‐quality wearable requirements. In the following section, we summarize the latest progress of elastic fibers/fabrics for energy harvesting/storage, sensors, and e‐skins, and the advantages of device elasticity are highlighted.

### 4.1 Energy Harvesting and Storage

Portable and wearable electronics have been indispensable for daily human life, and the energy management modules have attracted considerable attention as it is significant to ensure the continuous operation of the device.^[^ ^213^, ^214^, ^215^, ^216^, ^217^ ^]^ The module consists of an energy harvesting device as well as an energy storage module. The energy storage technologies are relatively mature compared to the portable energy harvesting devices. Nanogenerator is an emerging technology for distributed energy harvesting, which has gradually sparked a revolution in energy management for driving micro‐/nanoelectronics.^[^ ^151^, ^155^, ^184^, ^218^, ^219^, ^220^ ^]^ Based on different working mechanism, piezoelectric nanogenerator (PENG), triboelectric nanogenerator (TENG), and thermoelectric nanogenerator (TEG) exhibit respective advantages in device cost, power output, and operation environment.^[^ ^221^, ^222^, ^223^, ^224^, ^225^, ^226^ ^]^

**Figure 5:**
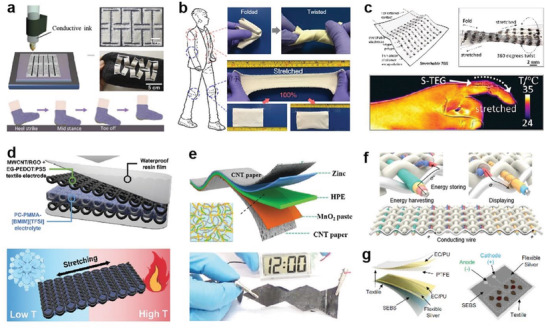
Elastic fibers based wearable devices for energy harvesting and storing. a) Fully 3D printed stretchable PENG mounted on a sock in a nonprotruding kirigami structure. Reproduced with permission.^[^ ^194^ ^]^ Copyright 2020, Elsevier. b) High performance and washable textile‐TENG with the capability to sustain various extreme deformation conditions. Reproduced with permission.^[^ ^203^ ^]^ Copyright 2018, Springer Nature. c) Stretchable TEG for thermoelectric energy harvesting from the dynamic surfaces of the human skin. Reproduced with permission.^[^ ^209^ ^]^ Copyright 2020, American Chemical Society. d) Stretchable high‐performance knitted fabric SC with temperature resistance. Reproduced with permission.^[^ ^210^ ^]^ Copyright 2021, Wiley‐VCH. e) A highly secure and portable flexible solid ZIB for powering portable electronics. Reproduced with permission.^[^ ^211^ ^]^ Copyright 2018, Royal Society of Chemistry. f) Energy harvesting and storage in an integrated system of fiber cells and solar cell fibers. Reproduced with permission.^[^ ^96^ ^]^ Copyright 2022, Springer Nature. g) A hybrid wearable e‐textile microgrid system based on BFCs and TENG for harvesting biochemical and biomechanical energy. Reproduced with permission.^[^ ^212^ ^]^ Copyright 2021, Springer Nature.

PENG is independent of operating frequency and humidity, which relies on piezoelectric materials that are usually hard or low‐flexible, achieving soft PENG to adapt to the deformation requirements of wearables well remains challenging.^[^ ^227^, ^228^, ^229^ ^]^ Zhou et al., using 3D printing, achieved a PENG with an elaborate kirigami structure, achieved high stretchability up to 300% without sacrificing electrical output, and the maximum power density of 1.4 µW cm^−2^ was achieved at a load resistance of 10^7^ Ω.^[^ ^194^ ^]^ As shown in **Figure** **5**a, being installed on a sock, the device smoothly collected voltage signals with various frequencies and accommodated the gaits while walking and stepping at different frequencies. TENG shows the merits of extensive material options and relatively higher power output.^[^ ^184^, ^230^, ^231^, ^232^ ^]^ Xiong et al. reported a wearable textile‐TENG through dip‐coating of black phosphorus and hydrophobic cellulose oleoyl ester nanoparticles (HCOENPs) on polyethylene terephthalate (PET) fabric, skin contacts can trigger the device to harvest biomedical energy.^[^ ^203^ ^]^ As shown in Figure 5b, the device showed instantaneous maximum output voltage and current density is 880 V and 1.1 µW cm^−2^, which maintained stable performance regardless of subjecting to extreme bending, twisting, stretching, and washing. It presents an energy harvesting strategy to simulate the practical wearing scenarios for sustained powering wearable electronics. TEG delivers good environment stability and is free of frequent maintenance. Yang et al. manufactured a stretchable TEG suitable for various complex and dynamic object surfaces (Figure 5c).^[^ ^209^ ^]^ The TEG mounted at the finger joint has good adhesion and can reliably collect energy from the natural temperature difference provided by human skin and the environment (output voltage change of ≈3% at a temperature difference of ≈5.5 °C). TEG provides a new option to utilize the heat energy around the human body for wearable energy harvesting. The development of energy harvestors presents an attractive future for distributed energy harvesting and self‐powered utilization, which could be an important supplement or replacement for traditional energy collection devices.^[^ ^233^ ^]^ To realize sustained energy utilization around the environment, energy storage modules with the matched electrical and mechanical properties should be developed to improve the all‐in‐one management of the harvested energy.

In recent years, textile materials (fibers, yarns, and fabrics) have been widely applied for energy harvestors (such as PENGs, TENGs, and TEGs) through different processing methods, diverse functional materials, demonstrating variant electrical output and power density. **Table** **2** summarizes the important works in this field and their respective features from processing to performance. Briefly, 1) fabric/textile‐based devices with larger effective area show apparently improved power density compared to fiber/yarn devices. 2) Yarn/fabric‐based devices present much higher stretchability than the fiber‐based devices due to their structural diversity. 3) Weaving/knitting is important for designing device patterns, improving the mechanical strength and durability of devices. 4) Functional nanocoating is effective for encapsulating devices, improving their washability and environmental stability.

**Table 2:** Materials and performances of energy harvestors based on fiber, yarn, and fabric

Device type	Device form	Fabrication	Materials	Parameters	Output performance	Strain [%]	Refs.
PENG	Fiber	Melt spinning	Carbon black, polyethylene, PVDF	1 cm, 10 Hz	23 nW cm^−1^	0.4	[234]
		Melt spinning	PI	0.3 N	219 mV	4.8	[235]
		Dipping	ZnO nanowire, PVDF	2 cm, <1.0 Hz	0.2 V, 10 nA cm^−2^, 2 µW cm^−3^	2.3	[236]
	Yarn	Triaxial braiding	PVDF, Ag, PA	2 cm, 0.25 Hz	380 mV, 29.62 µW cm^−3^	50	[157]
		Electrospinning	P(VDF‐TrFE)	1.87 g cm^−3^	16.2 mV	80	[158]
	Fabric	Weaving	PVDF, PET	4.95 cm^2^, 6 Hz, 6.6 MΩ	125 µW cm^−2^	20	[173]
		Knitting	PVDF, Ag, PA66	79.5 cm^2^, 0.106 kPa	14 V, 30 µA, 5.10 µW cm^−2^	–	[237]
		Dip‐coating	Glass fiber fabric, PDMS	64 cm^2^, 1 Hz	110 V, 16.41 nA cm^−2^	50	[238]
		3D printing	P(VDF‐TrFE), BaTiO_3_	1 cm^2^, 107 Ω, 60 N, 5 Hz	6 V, 2 µA cm^−2^, 1.4 µW cm^−2^	>300	[194]
		Electrospinning	MWCNT, P(VDF‐TrFE)	3 cm^2^, 10 MΩ, 1 Hz	18.23 V, 2.14 µA, 6.53 µW cm^−2^	>75	[239]
		Electrospinning	PU, P(VDF‐TrFE), BaTiO_3_, graphite	1 cm^2^, 1.0 Hz	9.3 V, 189 nA, 1.76 µW cm^−2^	40	[186]
TENG	Fiber	Dip‐coating	Cotton thread, CNT, PTFE	0.207 mg cm^−1^, 5 Hz	0.16 nC, 0.1 µW cm^−2^	2.15	[240]
		Coating	Silicon rubber, CNT, Cu	10 cm, 5 Hz	142.8 V, 0.18 µA cm^−1^, 6.1 nC m^−1^	70	[241]
	Yarn	Coating	PDMS, BaTiO_3_, SIS, PA	10 cm, 34 N, 10 Hz	76.8 V, 7.86 µA, 200.9 µW cm^−2^	250	[242]
		Coating	ZnS, PDMS	0.85 cm^2^	21 V, 0.1 µA, 8.5 nC	314	[156]
		Twisting	PI, PET, cotton	0.083 mg mm^−1^, 1000 MΩ, 30 N, 1 Hz	73.55 µW m^−1^	5	[108]
	Fabric	Weaving	Cu, PET, PI	Tapping speed: 10 cm s^−1^	4.98 V, 15.50 mA m^−2^	–	[109]
		3D orthogonal woven	Stainless steel, PET, PDMS	18 cm^2^, 132 MΩ, 3 Hz	45 V, 1.8 µA, 18 nC, 263.36 mW m^−2^	–	[243]
		Flat knitting	Cotton, PA, Ag, silicone rubber	25 cm^2^, 4 kPa, 4 Hz	1.2 V, 6.04 nA, 0.4 nC, 3.4 mW m^−2^	300	[110]
		Dip‐coating	PET fabric, HCOENPs, black phosphorus, Ag flakes, PDMS	49 cm^2^ 5 N, 4 Hz	880 V, 1.1 µA cm^−2^, 9 nC cm^−2^	100	[203]
		Dip‐coating	PET fabric, HCOENPs	2.25 cm^2^, water‐flow: 6 mL s^−1^, 100 MΩ	15 V, 4 µA, 0.14 W m^−2^	–	[219]
		Electrospinning,	SiO_2_, P(VDF‐TrFE)	Water‐flow: 10 mL s^−1^	36 V, 10 µA	>300	[117]
		Electrospinning, Electrospraying, Screen printing	PVDF‐HFP, SEBS, LM, Ag flakes	4 cm^2^, 5 Hz, 30 N, 20 MΩ	85 V, 4 µA, 15 nC, 219.66 mW m^−2^	490	[180]
TEG	Fiber	Electrospinning	CA, CNT	2 cm, Δ*T* = 41 K	65.4 mV cm^−2^	≈15	[244]
		Wet spinning	PEDOT:PSS	Δ*T* = 10 K	0.72 mV, 0.51 nW, 0.323 µW cm^−2^	30.5	[245]
		Electrospinning	CNT, PU, PEDOT:PSS	3 cm, *T* = 28 K, 3000 Ω	6.78 nW	350	[246]
	Yarn	Twisting	CNT, PDMS	21 cm^2^, Δ*T* = 25 K, *T* = 298 K	1.5 V, 1.2 mW	20	[168]
		Coating	PEDOT:PSS, Lycra	1.5 cm, Δ*T* = 95 K	1.3 mV, 480 pW, 0.5 µW	1000	[159]
	Fabric	3D weaving	CNT, PU	13.92 cm^2^, Δ*T* = 30 K	218 mV, 16.9 µW	100	[175]
		Knitting	CNT, PEDOT:PSS	Δ*T* = 44 K	700 µA, 4.64 µW, 70 mW m^−2^	80	[183]
		3D printing	Liquid metal, PDMS	1.848 cm^3^, Δ *T* = 60 °C	392 mV, 10.7 mW, 650 µW cm^−2^	30	[247]
		Electrospinning	PVP, PU, CNT	4 cm^2^, *T* = 24 °C	0.75 mV	250	[248]

The trend toward miniaturization and stabilization of innovative wearable electronics poses new requirements in improving energy storage devices’ power density and stability.^[^ ^249^, ^250^, ^251^ ^]^ Representative energy storage devices such as biofuel cells (BFCs), supercapacitors (SCs), and metal‐ion batteries have become mainstream because of their remarkable electrochemical properties.^[^ ^252^, ^253^, ^254^, ^255^, ^256^ ^]^ Efforts are devoted to improving their safety, integration, energy density, and environment adaptivity.

Realizing energy storage devices to adapt to complex conditions such as extreme temperature, humidity, and mechanical deformations is significant for practical application. For example, Lee et al. proposed a high‐performance textile‐based flexible SC (FSC) with mechanical and thermal stability.^[^ ^210^ ^]^ CNT/RGO+EG‐PEDOT coated a spandex/nylon knitted: PSS solution to act as an electrode, together with a gel‐type PC‐PMMA‐[BMIM][TFSI] electrolyte and resin encapsulation layer to assemble as the FSC. The device showed stable areal capacitances of 28.0, 30.4, and 30.6 mF cm^−2^ at different fabric (≈100% strain) temperatures (−30, 25, and 80 °C), which maintained stable performance after the experience of repetitive cooling–heating and 1000 cycles of stretching at 50% strain (Figure 5d). In addition, all‐solid‐state batteries that are generally considered safer than conventional lithium‐ion batteries could be realized with higher energy density.^[^ ^140^ ^]^ Li et al. fabricated a secure and wearable solid‐state zinc‐ion battery (ZIB) based on a hierarchically structured polymer electrolyte of gelatin and polyacrylamide (PAM) (Figure 5e).^[^ ^211^ ^]^ High‐areal energy density and power density (6.18 mW h cm^−2^ and 148.2 mW cm^−2^, respectively), high‐specific capacity (306 mA h g^−1^), and outstanding cycling stability (97% capacity retention after 1000 cycles at 2772 mA g^−1^) were demonstrated, stable performances can be well‐maintained even subjected various extreme conditions, such as cutting, bending, hammering, puncturing, sewing, as well as washing and firing, which can power electronic devices such as commercial electronic watches and smart insoles. Liao et al. innovatively proposed a mass‐producible three‐channel solution extrusion process to successfully prepare a three‐component fiber lithium electron cell (FLIB) consisting of parallel cathodes and anodes encapsulated by electrolytes.^[^ ^96^ ^]^ Considering the convenient integration of the fiber, through a rapier loom, the textile battery could be directly obtained by using FLIB as the weft and ordinary fiber material as the warp (Figure 5f). The energy density of a 10 m^2^ area textile battery is 550 mWh m^−2^. And the absolute discharge capacity of the textile battery is determined by the area of the fabric, which increases linearly when the area increases. Following international standards, it maintains good performance after 20 machine washings (<10% change in capacity), fully confirming the water resistance and stability of the device.

Integrating multiple devices for efficient energy harvesting and storage is promising to realize a more intelligent wearable energy management system.^[^ ^257^, ^258^, ^259^, ^260^ ^]^ It suggests that it can convert other forms of energy into electrical power and store it simultaneously, enabling more convenient and efficient management of energy devices for a range of applications. BFC is an energy harvestor from biofuel (such as sweat and tear); further improving its wearable application potential is attractive. Recently, Yin et al. designed a multimodule wearable bioenergy microgrid with nylon spandex fabric that can operate solely on human activities.^[^ ^212^ ^]^ The device consisted of a sweat‐based BFC and a TNEG, as shown in Figure 5g, the BFC can generate an electrical charge from lactic acid and oxygen in sweat. When exercising, the TENG would be activated from transient motion‐induced charge generation to harvest biomechanical energy to quickly launch the system, while the subsequently started BFCs harvest biochemical energy from electroenzymatic reactions of sweat metabolites for prolonged power delivery. SC could regulate the harvested biochemical and biomechanical energy to achieve high power output.

In short, although various wearable energy management devices have been exploited with improved performances, the wearable applications still require the devices to sustain the frequent deformations, which may cause device delamination, electrode damage or electrolyte leakage, leading to degradation of electrochemical performance, equipment failure, and even safety issues. Besides high‐power density, a future investigation should be focused on the innovation of materials and structures to improve mechanical stability, electrical reliability, and environmental durability, as well as the seamless integration of various energy harvestors and storages to improve the energy utilization efficiency and applicability.

### 4.2 Sensors and e‐Skins

Sensors are an irreplaceable part of an advanced wearable system that could monitor physiological information, perceive external objects and interact with machines or environments.^[^ ^261^, ^262^, ^263^, ^264^, ^265^, ^266^, ^267^, ^268^, ^269^, ^270^ ^]^ Wearable fiber/fabric sensors with high integration and mechanical compliance are promising to realize sensing with high sensitivity and fidelity, which are important for continuous monitoring and real‐time information transmitting, improving the feedback quality and human–machine–environment interactions.^[^ ^271^, ^272^, ^273^, ^274^ ^]^

**Figure 6:**
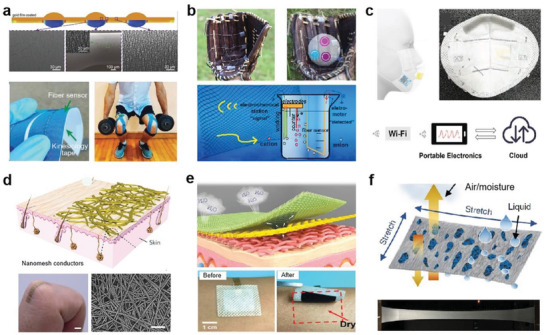
Elastic fibers based wearable sensors and e‐skins. a) Structured fiber strain sensors for reliable monitoring of sports activities. Reproduced with permission.^[^ ^275^ ^]^ Copyright 2017, Wiley‐VCH. b) A sensing network made of superelastic fibers for intelligent sports monitoring and ions detection. Reproduced with permission.^[^ ^93^ ^]^ Copyright 2021, Springer Nature. c) A smart face mask for wireless breath monitoring. Reproduced with permission.^[^ ^277^ ^]^ Copyright 2021, Wiley‐VCH. d) Noninflammatory and highly breathable stretchable e‐skin. Reproduced with permission.^[^ ^278^ ^]^ Copyright 2017, Springer Nature. e) Breathable, antibacterial, sweat‐stable e‐skin for bioelectric signal collection. Reproduced with permission.^[^ ^279^ ^]^ Copyright 2021, Wiley‐VCH. f) Elastic fibers conductor with excellent permeability, mechanical and electrical stabilities. Reproduced with permission.^[^ ^280^ ^]^ Copyright 2021, Springer Nature.

Liu et al. innovatively proposed a transient thermal curing method to design a fibrous stretchable strain sensor with inhomogeneous microstructure distribution.^[^ ^275^ ^]^ As shown in **Figure** **6**a, these microbeads can tune the strain distribution along with the fiber by rearranging the strain from the beads to the region between the beads, leading to a concentrated distribution of strain that could enhance the sensitivity. The fibrous sensor can be attached to the human body with tape or woven into the fabric for motion monitoring during exercise. Horev et al. developed SIS nanofibers based SIS‐PANI elastic sensing membrane, which can detect complex motion behaviors including bending and twisting with different angles (1°–180°) and deformation speeds (3–18 rpm). The ultrathin (300–10 000 nm) strain sensor could accurately judge different mechanical stimuli in a continuous way.^[^ ^276^ ^]^ In addition, by wrapping SEBS elastomer film on pillared PVA molds with different cross‐section shapes for subsequent thermal‐drawing treatment, Chen et al. demonstrated a two‐step soluble core method to prepare stretchable conductive fiber.^[^ ^93^ ^]^ The fiber can sustain ≈1900% strain and withstand a load of 1.5 kg load impact free‐falling from a height of 0.8 m. Owing to its excellent elasticity and mechanical compliance, the fiber woven as the 2D mesh was applied as a triboelectric self‐powered sensor for monitoring the impacting speed of baseballs (Figure 6b). Moreover, the waterproof fibers can also sense ion movements in various liquids, exhibiting multifunctional self‐powered sensing applications.

Recently, Zhong et al. developed a smart mask consisting of a self‐powered pressure sensor via electrostatic (triboelectric) effect.^[^ ^277^ ^]^ The sensor has the advantages of miniaturization (2.5 × 2.5 cm^2^), light weight (4.5 mg), and can output a peak voltage of up to 10 V during airflow stimulation. By wearing the mask as shown in Figure 6c, wireless monitoring of various breathing conditions such as normal breathing, shortness of breath, coughing, and breath‐holding can be realized, promising to record and analyze personal respiratory‐related diseases.

In addition to the wearable applications, soft electronics are also desired to be electronic skin (e‐skin) that generally consists of a sensor network and can be unobtrusively operated on human skin, which can detect and quantify various stimuli and deformations with responsiveness in real time to mimic the human somatosensory system.^[^ ^281^, ^282^, ^283^, ^284^, ^285^, ^286^, ^287^, ^288^ ^]^ E‐skin shows an important application for physiological detection, health monitoring, soft robots and human–machine interactions. One of the main challenges is the high requirements of e‐skin in properties such as air/moisture permeability, which are continually pursued to meet the long‐term on‐skin application. The device features such as ultrathinness, porosity, self‐adhesion, and self‐cleansing could effectively improve the application potential of e‐skins.

Most of the elastomer films applied for e‐skin devices have poor air/moisture permeability, which could not meet the long‐term on‐skin usage, avoid discomfort, and alleviate skin allergy or inflammation. All‐fiber structure to construct breathable e‐skin would be a promising route to tackle this concern.^[^ ^289^, ^290^ ^]^ Miyamoto et al. prepared an ultrathin breathable e‐skin with high stretchability and noninflammation (Figure 6d).^[^ ^278^ ^]^ The device was obtained by depositing an Au layer on the electrospinning PVA nanofilm that can be subsequently dissolved on skin in situ. The device showed high compliance and adhesion to the skin, which possesses excellent resistance stability (increases from 55 to 150 Ω) even after 10 000 cycles of finger bending with 40% elongation, demonstrating elastic on‐skin conductors with outstanding mechanical and electrical reliability.

Through modifying the cation and anion of poly(ionic liquid) (PIL) to adjust the chemical structure and properties, Zheng et al. prepared dual gradient PIL nanofiber films by electrospinning, demonstrating multilayer PIL films with air permeability, monohygroscopic function and antibacterial activity (Figure 6e).^[^ ^279^ ^]^ Unlike commercial gels that are unstable after sweating, the PIL film exhibited superior stability after 20 cycles of sweating–drying by artificial sweat, with no significant change in the detected ECG signal. The stability, comfort, and safety for long‐term use under sweating conditions, is essential for continuous and effective health monitoring. To further improve the deformability of breathable conductor, recently, Ma et al. screen‐printed liquid metal on an electrospinning poly(styrene‐*block*‐butadiene‐*block*‐styrene) fiber membrane, followed by a simple repetitive mechanical stretching to active the liquid metal layer and allow it penetrate to obtain a liquid‐metal fiber mat (LMFM) (Figure 6f).^[^ ^280^ ^]^ LMFM exhibits both superelasticity (above 1800% strain) and good permeability for gas/liquid, which can sustain more than 25 000 cycles at 100% strain and only shows ≈30% resistance change, indicating excellent mechanical and electrical stability enough for wearable applications.

In practice, the stability of electronic devices for operation under extreme conditions remained a huge concern. Undoubtedly, in addition to excellent electrical performance, wearable devices desire more practical functionalities, such as water‐resistance or washability,^[^ ^41^, ^109^, ^120^, ^184^, ^219^, ^291^, ^292^ ^]^ acid and alkali resistance and corrosion‐resistance,^[^ ^293^, ^294^, ^295^ ^]^ stain‐resistance,^[^ ^296^, ^297^ ^]^ diverse and large deformation stability,^[^ ^298^, ^299^, ^300^ ^]^ long‐term usage stability,^[^ ^301^, ^302^, ^303^, ^304^ ^]^ and so on. For instance, de Medeiros et al. developed a degradable omniphobic silk‐based coils (OSCs) utilizing silk proteins, CNT, and chitin carbon nanoflakes.^[^ ^305^ ^]^ The OSCs sewed into textiles can resist water, blood and most oils at room temperature. Wang et al. obtained strain sensors by carbonizing commercial silk fabrics, which can sustain 10 000 cycles of loading–unloading under 300% strain.^[^ ^306^ ^]^

In brief, fibers/fabrics for wearable applications, higher comprehensive properties in electrical output, power density, and sensitivity are important for promoting practical applications. The current technologies have presented promising strategies to push the boundaries of development. Meanwhile, new materials and technologies also emerged for continuous innovation of fiber and fabric materials, which could well meet the properties in mechanical compliance and permeability. For future development, multifunction derivation, integration, and a better balance of these performances in one device is significant for promoting fibers/fabrics for wearables.

## 5 Elastic Fibers/Fabrics for Bioelectronics

Bioelectronics represents a device that is directly in contact with living organisms and can transduce the stimuli from the body or environment into visible electrical signals or readable information, assisting the living organisms in realizing more intuitional perception, and feedback, response, and interactions. Elastic textiles could be directly applied for bioelectronics or be transplanted with the emerging device structures or technologies, achieving intelligent biointerfaces to function in thermal management, personal protection, wound healing, biosensing, and drug delivery. Establishing a good connection between the human body and these devices, achieving personalized wearables and medical care without time and space limitation is an important development tread for future bioelectronics.

### 5.1 Thermal Management

Personal thermal management is an emerging solution with the potential to improve human thermal comfort and reduce energy consumption for the daily required heating and cooling. Efforts have been devoted to functionalizing conventional textiles for efficient regulation of heat exchange with the surrounding environment.^[^ ^307^, ^308^, ^309^ ^]^

**Figure 7:**
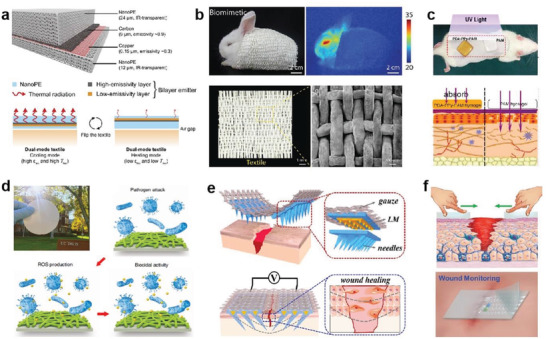
Elastic fibers‐based bioelectronics for thermal management, personal protection, and wound healing. a) Schematic diagram of the dual‐mode textile structure and its radiated emission. Reproduced with permission.^[^ ^314^ ^]^ Copyright 2017, The American Association for the Advancement of Science (AAAS). b) Thermally invisible textile woven from bionic porous fibers. Reproduced with permission.^[^ ^320^ ^]^ Copyright 2018, Wiley‐VCH. c) PDA‐PPy‐PAM hydrogels for UV shielding skin dressings. Reproduced with permission.^[^ ^149^ ^]^ Copyright 2018, American Chemical Society. d) A photograph of an RNM and its mechanism for releasing ROS for sterilization. Reproduced with permission.^[^ ^46^ ^]^ Copyright 2018, The American Association for the Advancement of Science (AAAS). e) Eagle's claw patch guided by electrical stimulation to repair wounds. Reproduced with permission.^[^ ^327^ ^]^ Copyright 2021, Elsevier. f) MFWD for wound closure, bacterial inhibition, and wound infection monitoring. Reproduced with permission.^[^ ^328^ ^]^ Copyright 2021, Wiley‐VCH.

In a relatively low‐cost and energy‐efficient manner, personal thermal management technology allows for the effective regulation of heat exchange between the human body and its surroundings, enabling applications in areas such as human thermal comfort, radiation insulation, and even military stealth.^[^ ^310^, ^311^, ^312^, ^313^ ^]^ Several innovative materials and assembly structures have been proposed to develop wearable thermally managed textiles. A typical example was demonstrated by Cui and co‐workers, they prepared a dual‐mode textile that can be heated and cooled without external energy, input by embedding a double‐layer emitter in nanoporous polyethylene (nanoPE).^[^ ^314^ ^]^ As shown in **Figure** **7**a, this double‐layer emitter consists of a high emissivity carbon layer and a low reflectivity copper layer. On the one hand, given the transparent nature of nanoPE to mid‐infrared human radiation,^[^ ^315^ ^]^ the embedded double‐layer emitter can freely conduct radiation to the environment. On the other hand, the choice of nanoPE (the fiber diameter of 50 to 1000 nm), water and moisture permeable as base material, also satisfies the requirements for breathability, comfort, and abrasion resistance. Simply by flipping the textile, the user has the option to adjust the emissivity and change the heat transfer coefficient easily to control both heating and cooling modes effectively. Besides, one of the primary approaches to fashioning textiles with tunable heat transmission properties is to coat the textile surface with thermally conductive materials, such as reflective materials of Ag, Ag NW, etc.^[^ ^316^, ^317^, ^318^ ^]^ For instance, Cai et al. coated nanoporous Ag film onto nanoPE modified with PDA in advance to obtain (nano‐Ag/PE).^[^ ^319^ ^]^ Further, cotton/Ag/PE can be prepared through lamination. Since the nano‐Ag/PE exhibits low infrared radiation (IR) emissivity of 10.1%, enabling the cotton/Ag/PE has an advantage over normal cotton fabrics in terms of body heating (up to 7.1 °C). These rational combination of functional materials with scalable coating methods could offer a sustainable route for personal management, global warming, and the energy crisis.

In addition, realizing thermal management through the accurate design of fiber structure is also an attractive solution. Inspired by polar bears that live in extremely cold regions, Cui et al. reported a “freeze‐spinning” method to produce fibers with aligned microporous structure using a mixed solution of silk fibroin, chitosan, and CNTs, which successfully imitated the structural features and functions of polar bear hair.^[^ ^320^ ^]^ Textiles woven from this biomimetic fiber, not only possess excellent thermal insulation properties for personal thermal management, but also are a good candidate for thermal camouflage materials, which can effectively function at the temperature range of −10 to 40 °C (Figure 7b). The rabbit wearing soft bionic textile is almost invisible under the infrared camera because its shell temperature is nearly close to the ambient temperature. Such materials provided inspiration for intelligent personal thermal management textiles as well as the exploitation of self‐adaptive infrared camouflage textiles, which are significant for both intelligent textiles for daily and military applications.^[^ ^312^, ^321^, ^322^ ^]^

### 5.2 Personal Protection

Since 2019, personal protective equipment (PPE) has been in undersupply worldwide for a while as the sudden globalization of the novel coronavirus pneumonia pandemic (COVID‐19) threatens public safety.^[^ ^323^ ^]^ PPE is crucial to protect front‐line healthcare workers who are in direct contact with patients from the aggression and infection of a number of diseases. The subject of developing ultrafiltration, antiradiation, antimicrobial, antiviral, and disinfection products to effectively inactivate germs and safeguard human lives has attracted increasing attention.^[^ ^46^, ^324^, ^325^, ^326^ ^]^ For example, Han et al. synthesized a PAM hydrogel with an in situ grown nanofibers network of polydopamine‐doped polypyrrole (PDA‐PPy‐PAM).^[^ ^149^ ^]^ PDA‐PPy‐PAM possesses good transparency, elasticity (>2000% strain) and strong adhesion In addition, PDA‐PPy that can absorb ultraviolet (UV) rays brings extra UV blocking capability, which can effectively protect the rat skin for injured under UV irradiation (365 nm, 30 mW cm^−2^, 20 min) (Figure 7c).

In addition, more attention was focused on autonomous antibacterial textiles to enrich the function of textiles in biosafety protection. Gong et al. prepared an MXene/PDA/Ni^2+^ (MPNi@Spandex) decorated spandex yarn by alternate dip‐coating using MXene nanosheets as “bricks” and PDA/Ni^2+^ as “mortar”.^[^ ^329^ ^]^ As a highly sensitive strain sensor, the yarn exhibits extra functions for thermal therapy and sterilization (99.9%) dependent on near‐infrared radiation. Si et al. modified PVA‐*co*‐PE electrospinning nanomembranes by the dip‐coating to fabricate a series of rechargeable antibacterial and antiviral nanofibrous membranes (RNMs).^[^ ^46^ ^]^ As shown in Figure 7d, RNMs can efficiently and continuously generate reactive oxygen species (ROS) to kill pathogenic microorganisms under light conditions. In terms of their photoactive features, RNMs have demonstrated their potential to be rechargeable for germicidal purposes through light irradiation. Further, RNMs enable convenient integration into medical masks and protective clothing, delivering interception effect on ultrafine particles and a bacterial and viral killer. Antimicrobial finishing agents are effectively applied to fight pathogens via killing viruses or inhibiting the living environment for viruses or bacteria.^[^ ^330^ ^]^ Compare to traditional static antibacterial textiles, the responsive antimicrobial materials are increasingly incorporated in fibers and textiles, delivering dynamic textiles with antibacterial responsiveness to external stimuli, effective to improve the environment adaptivity and extend the application scenarios of textiles. Increasing medical textiles would be derived to reduce the risk of exposure and cross‐contamination, providing enhanced PPEs for health protection and personal healthcare.

### 5.3 Wound Healing

Wound healing is a complex as well as a slow and dynamic process, accompanied by a series of concerns, such as slow recovery or unseemly healing resulting from chronic bacterial infection, which are still challenges that plague personal medical protection.^[^ ^331^, ^332^, ^333^, ^334^, ^335^ ^]^ New multifunctional wound dressings with smart responsiveness and dynamic adaptivity to construct nutritive microenvironment, promote cellular activity and accelerate wound healing exhibit fascinating potential.^[^ ^336^, ^337^ ^]^ Utilizing a top‐down multiple‐mold‐guided photolithography, Zhang et al. demonstrated a microneedle patch by mimicking the structure of the eagle's claw (Figure 7e).^[^ ^327^ ^]^ The microneedle patch was integrated by a medical gauze penetrated with the hydrogel. As a typical elastic textile, the medical gauze acts as an elastic substrate, which is important for function the device. Accordingly, benefiting from the claw‐shaped structure, the skin near the wound can be prevented from dehiscence and healed efficiently. In addition, the embedded LM could provide continuous electrical stimulation to the wound and efficiently promotes wound healing.

In addition, the presence of free ions, dipoles, and polarized molecules in the body allows cellular tissues and organs to generate electricity, and these tiny bioelectricities play a crucial role in health.^[^ ^338^ ^]^ Specifically, electrical stimulation demonstrates multiple medical effects, such as reducing edema, removing necrotic tissue, attracting neutrophils and macrophages, stimulating the growth of granulation tissue and inhibiting bacteria.^[^ ^339^, ^340^, ^341^ ^]^ Therefore, electrical stimulation therapy is already one of modern medicine's most vital treatment modalities. Following the mechanism, Long et al. created a wearable self‐activating electrotherapy bandage device, consisting of a nanogenerator and excipient electrodes.^[^ ^342^ ^]^ The nanogenerator was fabricated by Cu/PTFE layer (electronegative material) with another Cu layer (electropositive one) on different sides of a PET film, which can conformally attach on the mouse body and be triggered the respiration, generating electrical pulse with distinct strengths under shallow and strong breaching of the mouse. Animal studies indicated that the output electricity can accelerate the closure of a cutaneous wound within 3 days compared to the normal healing process of 12 days, which was attributed to the electric field‐facilitated fibroblast migration, proliferation, and transdifferentiation.

In general, wound healing is often assessed subjectively by the healthcare professional at the time of dressing changes. Aiming to acquire objective and visual information about the dynamic recovery of the wound, smart dressings to monitor the relevant information of the injured area in real time is interesting.^[^ ^343^, ^344^ ^]^ Tang et al. reported a multifunctional wound dressing (MFWD) that can dynamically monitor temperature, pH, and glucose levels in the wound area.^[^ ^328^ ^]^ First, the prepolymer PUIDE was successfully synthesized by condensing hydroxyl terminated polybutadiene, 1,10‐decanediol, and isophorone diisocyanate were catalyzed by dibutyltin dilaurate. Subsequently, PUIDE was mixed with cetyltrimethylammonium bromide (CTAB) solution to obtain PUIDE–CTAB elastomer by solution casting. PUIDE–CTAB not only exhibits excellent mechanical properties (7.34 MPa tensile strength and ≈1400% tensile strain), but also shows a self‐healing ability by virtue of the dynamic reassembly of hydrogen bonds. Figure 7f depicts that the MFWD is rationally composed of two PUIDE–CTAB films (as the substrates) and a sensing layer (including glucose, pH, and temperature sensors). While exerting antimicrobial power to create favorable wound recovery conditions, MFWD can provide information on the physiological changes in the wound to avoid wound infection and inflammation.

Various wearable devices with strategy innovations in material component, device structure, and working mechanisms are feasible for the promotion of wound healing. Fibers and fabrics with good breathability and comfortability provide more potential to construct more friendly and safe microenvironment. Further efforts to realize seamless integration of elastic fibers/fabrics with the emerging wound monitoring and healing technologies, promise to realize dynamic management and accelerate the development of modern medicine.

### 5.4 Biosensing

In order to break through the traditional mode of diagnosis and treatment that rely on relative unhandy instruments, the emerging point‐of‐care (POC) biosensing that enables instant and continuous monitoring of physiological information has become an essential component of modern smart healthcare services.^[^ ^345^, ^346^, ^347^, ^348^, ^349^ ^]^ A series of advances in sensing, imaging, wireless technology, IoT, and intelligent medical care are promoting the innovation and development of wearable bioelectronics technology at an alarming rate. The ability of biosensing to digitally and visually monitor vital signs and health parameters is increasingly important in disease management.^[^ ^350^, ^351^, ^352^, ^353^ ^]^ By being integrated into textiles, placed on the skin, or implanted in the body, these devices can collect information in real‐time and interact effectively with terminal devices or administrators, gradually enabling miniaturization, portability, noninvasiveness, accuracy, and stabilization become possible. Depending on working mechanism, the sensors for physiological information monitoring can be generally classified as physical sensing and chemical sensing.^[^ ^349^, ^354^ ^]^

**Figure 8:**
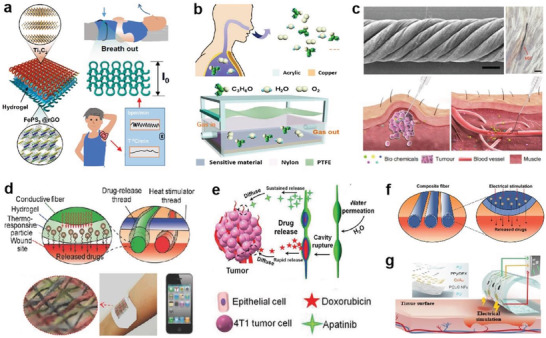
Intelligent biosensing devices and advanced DDSs. a) Cotton fabric‐based wearable platform for respiration and body temperature monitoring. Reproduced with permission.^[^ ^356^ ^]^ Copyright 2022, Springer Nature. b) Self‐powered wireless acetone sensors for noninvasive diabetes detection. Reproduced with permission.^[^ ^362^ ^]^ Copyright 2020, Elsevier. c) MSF sensor injected into the body for abnormal index detection. Reproduced with permission.^[^ ^369^ ^]^ Copyright 2019, Springer Nature. d) A core–shell conducting fiber for transdermal drug delivery. Reproduced with permission.^[^ ^370^ ^]^ Copyright 2017, Wiley‐VCH. e) An implantable fiber device capable of releasing combination drugs for synergistic therapy. Reproduced with permission.^[^ ^371^ ^]^ Copyright 2019, Wiley‐VCH. f) PSAP microfibers release drugs under electrical stimulation. Reproduced with permission.^[^ ^148^ ^]^ Copyright 2022, Wiley‐VCH. g) A smart array patch for physiological signal detection and drug release. Reproduced with permission.^[^ ^372^ ^]^ Copyright 2022, Elsevier.

Physical sensing relies on transformation of quantities such as displacement, strain, pressure, temperature, etc. to measurable electrical signals. Qaiser et al. designed a piezoresistive sensing system consisting of a single‐walled CNT‐based POC strain sensor.^[^ ^355^ ^]^ The device with stretchability higher than 100% can firmly fit the skin, providing real‐time monitoring of throat‐related diseases such as the swelling of the salivary parotid glands caused by flu and mumps. When there is local swelling around the mandible‐ear region, the sensor can provide the history of swelling or the speed of muscle swelling and transmit the data to the smartphone for further diagnosis and treatment. Vaghasiya et al. coated FePS_3_@rGO and Ti_3_C_2_ on cotton fabric to construct a stretchable asymmetric supercapacitor (SASC).^[^ ^356^ ^]^ The SASCs can be connected in series with strain sensors and temperature sensors, acting as a low‐cost and portable platform for health monitoring (**Figure** **8**a). Respiratory syndrome and body temperature can be synchronously monitored in real‐time.

Chemical sensing often refers to monitoring the concentration of biological metabolites (sweat, tears, hormones, etc.) to reflect the physiological state of subjects. More than 500 volatile organic compounds may be detected in human breath during the metabolic process.^[^ ^357^ ^]^ Thanks to them, studies related to analyzing the gas composition of breath have been applied to noninvasive medical evaluation and disease diagnosis.^[^ ^358^, ^359^, ^360^, ^361^ ^]^ For example, a direct correlation exists between blood glucose concentration in exhaled breath and acetone concentration. Based on the sensitivity of chitosan (CS) and reduced graphene oxide (RGO) to acetone gas, Su et al. adopted a spray method to prepare CS–RGO sensing films to develop a wearable self‐powered acetone sensor (WSAS) for real‐time respiratory monitoring and diabetes diagnosis.^[^ ^362^ ^]^ The WSAS is integrated from the gas sensing and energy harvesting parts (Figure 8b). PTFE and PA, with opposite frictional electrical properties, successfully achieve the effect of wireless power transmission by generating electrical currents through contact with vibrations caused by breathing air currents. The CS‐RGO film presents a good sensing response (27.89%) at a concentration of 10 ppm acetone, which is ≈5 times higher than pure CS film. As a noninvasive health detection and monitoring platform, it offers new ideas for modern medical disease diagnosis and real‐time continuity.

Applying implantable electronics in the human body for biomedical research has become a hot topic of considerable interest. Compared to 2D and 3D shapes, it is apparent that 1D fiber shape electronics offer unique advantages (ease of integration, flexibility, lightness, etc.) for wearable and implantable applications.^[^ ^363^, ^364^ ^]^ Diverse implantable fiber electronics (sensors, batteries, and so on) enable in vivo disease prevention, continuous real‐time monitoring, and disease treatment, which play an important role in developing intelligent modern medicine.^[^ ^365^, ^366^, ^367^, ^368^ ^]^ According to bionics, Wang et al. twisted CNT fibers into spiral fiber bundles similar to the hierarchical structure of muscle fibers. They processed them into an implantable electrochemical sensor with a favorable match with tissues.^[^ ^369^ ^]^ Specifically, the CNTs are modified to endow them with various functions, resulting in single‐ply sensing fibers (SSFs) (Figure 8c). Further, the SSFs are twisted to yield multiply sensing fibers (MSFs). In combination with the injection technique, the MSFs are smoothly and accurately implanted into the tumor cells or blood vessels. The end of the MSF is maintained outside the body for subsequent analysis (hydrogen peroxide levels in tumor cells, ion concentrations, and glucose concentrations in the blood, etc.). They found that the MSF did not trigger infection or blood flow fluctuations during the 5 days in the experimental cats, and the wounds healed within 2 days after the MSF was removed. This research proposed a new research direction of injectable electronic devices that could balance mechanical compliance.

In short, the rapid development of biosensing devices provides more options and possibilities for noninvasive monitoring and evaluating human health status, promoting effective diagnosis of a specific disease. It promises to realize continuous physiological monitoring independent of time and space, such as personal healthcare for the disabled at home, which could largely relieve the burden of health care.

### 5.5 Drug Delivery and Therapy

In addition to continuously personal healthcare, the instant diagnosis and treatment raised more attention and interest for the on‐demand personalized medicine.^[^ ^373^ ^]^ Drug delivery system (DDS) is a well‐developed medical tool for precise drug release by controlling the dosage and administration interval.^[^ ^374^, ^375^, ^376^ ^]^ Typically, the DDS can be generally classified as transdermal, oral, spray, injection, and implantable administrations.^[^ ^377^ ^]^ Fiber materials with tunable geometrics and functions in a wide range, could act as a drug carrier for transdermal, as well as oral and implantable DDSs.^[^ ^378^ ^]^

Mostafalu et al. constructed functionalized cotton fibers by coating carbon ink and hydrogel of alginate/poly(ethylene glycol) diacrylate (PEGDA) with drug‐loaded heat‐sensitive poly(*N*‐isopropylacrylamide) (NIPAM) particles, and demonstrated them as fabric‐dressing for wound healing (Figure 8d).^[^ ^370^ ^]^ The carbon ink coating provided electrical conductivity for the cotton threads to construct micro heaters. Typically, setting the temperature to the critical dissolution temperature of PEGDA/NIPAM (40 °C) triggers a conversion from a hydrophilic/swelling phase to a hydrophobic/contracting phase, which allows independent release of multiple drugs and precise control of their temporal release rates. In a scratch test simulating cell migration during wound healing, the fabric‐dressing reduced the scratch width to 20% after 48 h compared to the dressing without drug, confirming its effectiveness in controllable transdermal drug release.

In addition, fibers are also feasible in in vivo drug delivery. By using a novel microfluidic triaxial electrospinning technology, Li et al. manufactured an implantable fiber device with a three‐layer structure (glycerol‐Dox/PLA/PCL‐Apa) loaded with the hydrophilic chemotherapeutic drug of doxorubicin hydrochloride (Dox) (inner layer) and hydrophobic tumor vascular inhibitor apatinib (Apa) (outer layer) for breast cancer treatment (Figure 8e).^[^ ^371^ ^]^ The device allows a combined release of the two drugs in vivo, the release rate can be effectively adjusted by tuning the concentration of the PLA solution (middle wall layer). For example, when the concentration is reduced to make thin walls of the inner cavity, rapid Dox release can be achieved, while Apa in the outer layer is slowly released through the slow degradation of PCL matrix. In this way, the controllable drug dosage can be delivered to realize local treatment in a synergistic therapeutic effect. By electrospinning of PU with superparamagnetic graphene oxide (SPGO), Sasikala et al. prepared an implantable anticancer device (SPGO nanofiber film, SPGO NF) for the treatment of breast cancer, enabling drug release, wound reconstruction and noninvasive detection.^[^ ^379^ ^]^ Thanks to the ability of SPGO to induce heating, the device is able to carry out thermal therapy to effectively reduce the risk of local recurrence. Utilizing the different degrees of hydrogen bonding interactions between SPGO and Dox at different pH conditions, Dox can be activated at tumor pH condition to realize continuous release over 60 days.^[^ ^380^ ^]^ The biocompatible electrospinning PU nanofiber matrix can influence adipogenesis and considerably support soft tissue reconstruction.^[^ ^381^ ^]^

Electrical control is an accurate method that is mainly used for device manipulation. To realize more precise release of drug in fibers, Xiong et al. demonstrated a poly(3,4‐ethylenedioxythiophene):polystyrene sulfonate (PEDOT:PSS)‐based microfluidic‐spun microfiber for controllable release of acetaminophen via electrical stimulation (Figure 8f).^[^ ^148^ ^]^ The fiber consisting of polyvinyl pyrrolidone/sodium alginate/acetaminophen/poly(3,4‐ethylenedioxythiophene):poly (styrene sulfonate) (PVP/SA/AAP/PEDOT:PSS, PSAP) shows core–shell structure with surface microcracks, which can be stimulated by electricity to realize accelerated drug diffusion and local concentration difference, promoting the drug release. The work presents an accurate fabrication strategy of fibers‐based patches with different geometrics for controllable drug release, it provides potential material option for wearable bioelectronics and self‐powered on‐demand DDSs.

Huang et al. invented a flexible smart array patch with a drug release and a strain sensor module for in vivo and ex vivo applications.^[^ ^372^ ^]^ The patch consisting of an aqueous PU substrate, a polycaprolactone‐gelatin electrospinning nanofiber film, as well as a patterned Au electrode and a PPy layer loaded with dexamethasone (DEX) (Figure 8g). The resistive responsive patch demonstrates excellent elasticity and skin compliance for sensitive monitoring of body signals. Thanks to the porous structure of fiber membrane, the loaded DEX shows a high release value of 54.9 µg cm^−2^ at a safe low voltage of 0.6 V within 1 min. This integrated patch demonstrates potential for cardiac signal detection and synchronous drug release, representing an important development direction of intelligent POC.

Compared to the extra electricity infliction, self‐powered devices such as TENG that can convert biomechanical activities into electricity, exhibit significant potential in exploiting self‐powered DDS platform.^[^ ^382^ ^]^ For example, one of the main concerns in the eye drops administration is that large lack of drug accompanied by tears and eyelid movement.^[^ ^383^ ^]^ Therefore, increasing the utilization and half‐life of drugs in the eye is important for treating eye diseases with higher efficiency and fewer side effects.^[^ ^384^ ^]^ Song et al. reported the first TENG‐based self‐powered implantable drug delivery system, the TENG can harvest human motion energy to drive an electrochemical microfluidic pump to realize drug delivery.^[^ ^385^ ^]^ The drug‐delivery speed depends on the gas production rate in electrolysis, which is determined by the current output from TENG. Typically, the iDDs have achieved pumping flow rates from 5.3 to 40 µL min^−1^ triggered by TENG. A 1 mm incision was made in the sclera, and the device was implanted in the eye, demonstrating a self‐powered control of drug delivery for potential ocular diagnosis. Ouyang et al. proposed the concept of TENG‐based self‐powered system for on‐demand transdermal drug delivery.^[^ ^386^ ^]^ The DDS consists of transdermal patch electrodes (drug patch and iontophoresis patch), a miniaturized TENG and a power management circuit with a model drug of DEX sodium phosphate (DEX‐P). The TENG converted biomechanical energy into electricity to provide electrical stimulation, enabling drug release from the conductive PPy film. Meanwhile, the TENG further functioned to activate the iontophoresis patch to improve the drug delivery in the skin. Drug release rates can reach doses of 0.05 to 0.25 µg cm^−2^ min^−1^, meeting the needs of on‐demand dosing. In addition, in vitro experiments also demonstrated that the system improved the drug delivery efficiency by ≈50% over conventional transdermal patches. This self‐powered on‐demand DDS system promises to greatly broaden the development of personalized healthcare and provide new approaches for efficient diagnosis and treatment.

Textile materials (fibers, yarns, fabrics) have been widely applied in energy harvestors and DDS platforms, however, self‐powered DDS platforms remains at the early stages of research.^[^ ^387^ ^]^ As favorable wearable or bio‐interface carriers, textile materials with diverse geometrics and accurate capability for loading and releasing objects provides great potential to incorporate the transdermal or implantable drug delivery technologies, realizing fiber/fabric‐based DDSs with capabilities for target drug delivery as well as self‐powered drug delivery. These electrically controllable intelligent DDS platforms with good wearability and skin affinity would largely promote the development of personalized healthcare and diagnosis.

## 6 Conclusions and Perspectives

Flexible electronics triggered a drastic technological revolution to exploit wearables and bioelectronics for constructing more intelligent platforms for energy and information interaction between organisms, objects, and the surrounding environment. Elastic fibers/fabrics with unique mechanical softness and structure superiority can assist electronic devices in improving their mechanical compliance and functional permeability to build operation‐friendly and biocompatible interfaces between the body, skin, organs, plants, and machines, offering the possibility of energy harvesting, information interaction, health monitoring and disease detection, as well as intervention therapy. Important progress has been achieved in improving the stretchability, sensitivity, biofriendliness, electrical output, and power density properties of e‐textiles, for instance, in the development of superelastic fiber/fabric sensors for real‐time monitoring of complex movements and, in conjunction with fiber/fabric TENG, for energy conversion, storage, and utilization to ensure the long‐term operation of e‐textiles.

**Figure 9:**
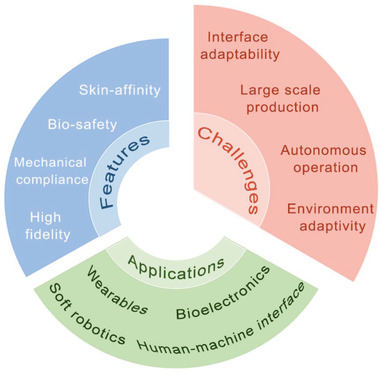
Challenges of elastic fibers/fabrics for wearables and bioelectronics.

However, the existing products and development methods do not highly match the practical demands of the applications in terms of industrial‐scale manufacturing, high electrical output, signal sensitivity and fidelity, diverse environment adaptivity, and multiscenarios on‐spot applications. For fiber/fabric‐based wearables and bioelectronics, the core issue is the material and technology limitations that could not achieve effective integration for device components, good interface adaptivity between components as well as between device and substrate, which is crucial for continuous device operation with high stability, sensitivity, and high fidelity (**Figure** **9**).

i) Device to adapt large‐scale production. The majority of the reported electronic textiles with great performance for wearables or bioelectronics are produced using laboratory precision design, the materials and fabrication processes should be improved to adapt to the high‐speed and high‐strength conditions in industrial scale production. Multifunctional nozzles with well‐designed structures to combine the extruding, thermal drawing technologies could provide more possibilities to create fiber/fabric devices in a more accurate and efficient solution.
ii) Interface adaptability of device to substrates. Repeated device operation inevitably causes delamination between the functional layers, leading to performance degradation, signal distortion, or even safety issues. Improving interface adaptability of electrode materials to the fiber‐based substrates is important, such as Young's modulus matching, wettability modification, electrical compensation, and interface adhesion enhancement. Printable conductive materials with self‐attachability, self‐healing, and dynamic electrical stability would be one of the important directions to tackle the challenges.
iii) Autonomous continuous operation at different scenarios. Organisms with diverse structures, mechanics, and physiological features require interface devices with high adaptivity for different systems. Extreme environments call for functions such as hydrophobicity, thermal/chemical resistance, UV resistance, flame retardancy, and self‐healing ability. In addition, the sustained power supply is critical for the device's long‐term application. The device capable of distributed energy management is promising to realize self‐powered functional devices and continuous on‐spot applications for different scenarios.

In addition to integrating the features above, the chemical, mechanical, optical, and electrical properties of the fiber/fabric selected raw materials should be involved in a deep discussion. The evolution of materials innovation and technologies will bring bright prospects for creating electronics based on elastic fibers/fabrics, delivering breathable electrical platforms for biointerface interactions with bioaffinity, friendliness, and biocompatibility, embracing the future development of wearables and bioelectronics.

## Conflict of Interest

The authors declare no conflict of interest.
